# Systematic Review: Quantitative Susceptibility Mapping (QSM) of Brain Iron Profile in Neurodegenerative Diseases

**DOI:** 10.3389/fnins.2021.618435

**Published:** 2021-02-18

**Authors:** Parsa Ravanfar, Samantha M. Loi, Warda T. Syeda, Tamsyn E. Van Rheenen, Ashley I. Bush, Patricia Desmond, Vanessa L. Cropley, Darius J. R. Lane, Carlos M. Opazo, Bradford A. Moffat, Dennis Velakoulis, Christos Pantelis

**Affiliations:** ^1^Melbourne Neuropsychiatry Centre, Department of Psychiatry, The University of Melbourne and Melbourne Health, Carlton South, VIC, Australia; ^2^Neuropsychiatry, The Royal Melbourne Hospital, Parkville, VIC, Australia; ^3^Centre for Mental Health, Swinburne University of Technology, Hawthorn, VIC, Australia; ^4^Melbourne Dementia Research Centre, Florey Institute of Neuroscience & Mental Health, The University of Melbourne, Parkville, VIC, Australia; ^5^Melbourne Brain Centre Imaging Unit, Department of Medicine and Radiology, The University of Melbourne, Parkville, VIC, Australia; ^6^Department of Radiology, The Royal Melbourne Hospital, The University of Melbourne, Parkville, VIC, Australia; ^7^Florey Institute of Neuroscience and Mental Health, The University of Melbourne, Parkville, VIC, Australia

**Keywords:** quantitative susceptibility mapping, brain, iron, Alzheimer's disease, Parkinson's disease, neurodegenerative diseases

## Abstract

Iron has been increasingly implicated in the pathology of neurodegenerative diseases. In the past decade, development of the new magnetic resonance imaging technique, quantitative susceptibility mapping (QSM), has enabled for the more comprehensive investigation of iron distribution in the brain. The aim of this systematic review was to provide a synthesis of the findings from existing QSM studies in neurodegenerative diseases. We identified 80 records by searching MEDLINE, Embase, Scopus, and PsycInfo databases. The disorders investigated in these studies included Alzheimer's disease, Parkinson's disease, amyotrophic lateral sclerosis, Wilson's disease, Huntington's disease, Friedreich's ataxia, spinocerebellar ataxia, Fabry disease, myotonic dystrophy, pantothenate-kinase-associated neurodegeneration, and mitochondrial membrane protein-associated neurodegeneration. As a general pattern, QSM revealed increased magnetic susceptibility (suggestive of increased iron content) in the brain regions associated with the pathology of each disorder, such as the amygdala and caudate nucleus in Alzheimer's disease, the substantia nigra in Parkinson's disease, motor cortex in amyotrophic lateral sclerosis, basal ganglia in Huntington's disease, and cerebellar dentate nucleus in Friedreich's ataxia. Furthermore, the increased magnetic susceptibility correlated with disease duration and severity of clinical features in some disorders. Although the number of studies is still limited in most of the neurodegenerative diseases, the existing evidence suggests that QSM can be a promising tool in the investigation of neurodegeneration.

## Introduction

While the presence of iron is vital for normal function and development of the brain, excess iron deposition has been proposed to play an important role in the pathology of neurodegenerative diseases (Morris et al., 2018). Iron is a key element in several metabolic pathways throughout the body including oxidative phosphorylation and DNA synthesis and is responsible for oxygen transport in the blood. In the central nervous system (CNS), in addition to its general metabolic roles, iron plays a critical part in myelin synthesis and neurotransmitter production (Mills et al., 2010). On the other hand, there has been a growing body of evidence in recent decades suggesting a role for iron in the pathology of neurodegenerative diseases, such as Alzheimer's disease (AD), Parkinson's disease (PD), and Huntington's disease (HD) (Masaldan et al., 2019).

The proposed role of iron in neurodegeneration is mediated through two mechanisms. In the first mechanism that can occur in all individuals regardless of illness, iron induces oxidative damage through production of reactive oxygen species and orchestrates “ferroptosis,” a recently identified form of iron-mediated cell death (see more about ferroptosis at Dixon et al., 2012) (Ndayisaba et al., 2019). The second mechanism is more disease-specific. Iron has been shown to interact with hallmark features of neurodegenerative illnesses, such as amyloid-β (Aβ) plaques, α-synuclein aggregates, and tau protein. The interplay between iron and these proteins promotes their production and aggregation, and incorporation of iron in their structure further increases the oxidizing capacity resulting in neuronal cell death (Masaldan et al., 2019; Ndayisaba et al., 2019).

For the reasons mentioned above, *in vivo* evaluation of brain iron has been of great interest in neurodegenerative diseases. Magnetic resonance imaging (MRI) can detect iron due to its high magnetic susceptibility. Magnetic susceptibility is a dimensionless physical property that indicates the magnetizability of a material when exposed to an applied magnetic field. Elements and compounds are categorized as paramagnetic or diamagnetic based on their magnetic susceptibility. Paramagnetic substances (such as most biologic forms of iron and copper) have a positive magnetic susceptibility, are attracted to an external magnetic field, and increase the mean tissue magnetic susceptibility. On the other hand, diamagnetic substances (such as water, myelin, and calcifications) have a negative magnetic susceptibility, are slightly repelled by an external magnetic field and decrease the mean tissue magnetic susceptibility (Liu et al., 2015; Rumble, 2020).

Mean magnetic susceptibility of organic tissues is determined by their composition and the magnetic susceptibility of their constituents. The brain tissue is generally weakly diamagnetic since water (slightly diamagnetic) constitutes 70–85% of the brain. Magnetic susceptibility varies slightly among brain regions due to the differences in their tissue composition. The major contributors to the measurable changes of magnetic susceptibility across the brain are myelin (weakly diamagnetic) and iron-containing molecules (mostly strongly paramagnetic). The largest proportion of non-heme iron in the brain is bound to ferritin, which makes up the greatest contribution to the tissue magnetic susceptibility among all iron compounds. Other forms of iron, including free and transferrin-bound iron only minimally contribute to the measured mean tissue susceptibility (Liu et al., 2015; Deistung et al., 2017; Duyn and Schenck, 2017). Another iron-containing complex that is abundant in the substantia nigra pars compacta (SNc) and locus coeruleus and constitutes a main source of magnetic susceptibility in these areas is neuromelanin. Neuromelanin is a dark pigment with a structure similar to melanin found in the skin and iris. It is produced from the oxidation of DOPA and dopamine and has high affinity and capacity for chelation of iron and other metals, constituting a major iron storage site in the catecholaminergic ganglia (Haining and Achat-Mendes, 2017).

MRI sequences that are sensitive to tissue magnetic susceptibility are used for investigation of iron. The contrast in susceptibility-based MRI techniques, such as susceptibility weighted imaging (SWI), ${{\text{T}}_{2}}^{*}$-weighted imaging (quantifying effective transverse relaxation), ${{\text{R}}_{2}}^{*}$ imaging (reciprocal of ${{\text{T}}_{2}}^{*}$), and quantitative susceptibility mapping (QSM), arises from microscopic magnetic field shifts due to the variations of tissue magnetic susceptibility. Recently developed QSM techniques provide quantitative estimates of local magnetic susceptibility at a voxel-level (Deistung et al., 2017). In each voxel, the local magnetic field that is used to calculate susceptibility is comprised of the background field (magnetic field from sources outside the brain or even outside the scanner), the magnetic field from neighboring voxels, and the local magnetic field produced by the tissue within the voxel. The main strength of QSM compared to traditional susceptibility-sensitive imaging techniques is that it disentangles the local magnetic field from the non-local contributions by solving a complex field-to-source inversion problem (Haacke et al., 2015). Advanced inference techniques enable QSM to localize, quantify, and produce a voxel-wise mapping of mean tissue susceptibility (Haacke et al., 2015; Wang and Liu, 2015).

In the deep gray matter structures, due to less confounding effect from myelin and negligible contribution from other paramagnetic metals, QSM has been shown to reliably quantify changes of iron content (Langkammer et al., 2012). The accuracy of QSM in identifying iron deposition in these regions has been validated in post-mortem studies showing significant correlations between QSM contrast and histochemical measurement of iron (Langkammer et al., 2012; Sun et al., 2015; Hametner et al., 2018; Lee et al., 2018; Lewis et al., 2018; Wang et al., 2020). In the white matter, on the other hand, alterations in magnetic susceptibility measured by QSM may result from changes in myelin as well as iron. In other words, in the basal ganglia, an increased magnetic susceptibility is most likely arising from an increase in iron content (except in the conditions where other paramagnetic metals are also increased), while such increase in the white matter can be a result of both an increase in iron, a decrease in myelin (demyelination) or both (Hametner et al., 2018).

Over the past decade, QSM has been used by an increasing number of studies in investigations of brain changes in neurodegenerative disorders. In this paper, we systematically reviewed existing human studies that investigated brain changes in neurodegenerative diseases using QSM. It is important to note that QSM does not directly measure iron content, rather it provides an accurate measurement of tissue magnetic susceptibility. To determine whether the alterations in magnetic susceptibility are indicative of changes in iron content, the brain region where such changes are observed (white vs. gray matter), as well as the pathophysiologic processes involved in each neurodegenerative disease should be taken into account. Therefore, in reporting the findings from QSM studies, we present the direct measures made by QSM, which is tissue magnetic susceptibility. In the discussion section, we will provide the implications of the findings in terms of brain iron changes in each neurodegenerative disease separately.

The overarching aim of this review was to determine regional disease-specific patterns of brain iron distribution in these disorders. To this end, we sought to address the following questions:

- Are there any differences in brain tissue magnetic susceptibility in patients with neurodegenerative diseases in comparison to healthy individuals?
- In patients with neurodegenerative diseases, do the regional changes of magnetic susceptibility correlate with the clinical manifestations of the disease and the areas that are most affected in these conditions?
- Is there evidence that the pattern of magnetic susceptibility changes identified by QSM can differentiate neurodegenerative diseases?

## Methods and Materials

### Search Protocol

This review followed the Preferred Reporting Items for Systematic Reviews and Meta-Analyses (PRISMA) guidelines (Moher et al., 2009; Shamseer et al., 2015). Our protocol was registered at the International Prospective Register of Systematic Reviews (https://www.crd.york.ac.uk/prospero/) (CRD42020168598) and was also published as a preprint at the publicly available domain, MedRXiv (Ravanfar et al., 2020).

We searched MEDLINE (PubMed interface), Embase (Ovid interface), Scopus, and PsycInfo (Ovid interface) databases. To ensure the inclusiveness of our search, we examined the reference lists of reviewed studies and relevant literature reviews to identify any further relevant records. Literature search strategies were developed to identify any record containing QSM or “quantitative susceptibility mapping” and any disease name under the Medical Subject Headings (https://www.ncbi.nlm.nih.gov/mesh/) subheading for “neurodegenerative diseases” in their title, abstract and keywords. Details of our search strategy can be found in our systematic review protocol, provided in Supplementary Material 1. The initial search was performed in January 2020 and amended in April 2020 to include all studies published during the time of preparing the manuscript.

### Study Selection

Authors PR and SL independently screened the records for inclusion according to the eligibility criteria specified below. In the first stage, titles and abstracts were screened and studies that met our eligibility criteria were included. Full text records of the studies included in the first stage were obtained and screened in the second stage for final decision on inclusion or exclusion. The two reviewers discussed any disagreements and if a consensus could not be reached, a third reviewer (CP) adjudicated the case. Studies included in this review were human studies published as journal articles in English without any limitation of publication date, according to the following criteria.

#### Design

We included cross-sectional and longitudinal case control studies, retrospective and prospective. Case reports and case series were not included.

#### Participants

We included studies investigating patients who were formally diagnosed with any of the disorders recognized as a neurodegenerative disease according to the National Institute of Health, Medical Subject Headings (MeSH), regardless of age. It should be noted that although a large number of QSM studies in multiple sclerosis exist, since the disorder is not classified as a neurodegenerative disease in MeSH, it has not been included in this review [Studies of QSM in individuals with MS have been reviewed elsewhere (Lee et al., 2017)].

Only those studies that presented the results from an independent sample of data were included. If a study population was used in more than one study, only the first published study was included provided that it satisfied other eligibility criteria.

#### Investigations

Of interest to this review were the studies investigating the brain structure in neurodegenerative diseases using QSM imaging with MRI scanners of any field strength. Studies that used other susceptibility-based imaging techniques, such as SWI, ${{\text{T}}_{2}}^{*}$, and ${{\text{R}}_{2}}^{*}$ without QSM were excluded.

#### Comparators

Included studies were those that:

- compared QSM findings between each neurodegenerative disease and healthy controls and/or,
- compared different subtypes or clinical features of a disease and/or,
- examined the relationship of QSM indices with pathologic markers of the disease.

Studies that focused on the QSM protocols and technical aspects using a population of either healthy participants or patients without any comparison between patients and healthy groups were excluded.

#### Outcomes

The outcome measure of interest for this review was magnetic susceptibility reported in ppm (parts per million) or ppb (parts per billion). In some studies, mean susceptibility values of the brain regions of interest (ROIs) were not reported, but the results from inter-group comparisons and correlation analyses were provided. This was not considered as an exclusion factor.

### Data Extraction, Synthesis, and Quality Assessment

Relevant information was extracted and recorded in summary tables for each study by PR and SL independently and compared for discrepancies. Quality assessment was performed by evaluating the risk of bias in each study using a modified version of the National Heart, Lung, and Blood Institute quality assessment tool for case-control studies which was customized to improve its application to our target studies (Supplementary Material 1). Factors, such as unclear description of study population and eligibility criteria, data processing by researchers who were not blinded to the clinical characteristics of subjects, inconsistent MRI acquisition and/or QSM processing, and failure to account for confounders, such as age in the statistical analysis, were considered to pose a risk of bias and reduce the quality of the study. Based on these factors, the risk of bias was reported as “high,” “medium,” and “low” for each study. The data extraction table and quality assessment tool are available in the systematic review protocol (Supplementary Material 1).

Due to the different methods of MRI acquisition, QSM processing, brain segmentation, and QSM reference regions among the reviewed studies, a meta-analysis was not possible. However, in an effort to quantify the magnitude of magnetic susceptibility differences between patient and healthy control groups reported by each study, we calculated the effect size (Hedge's *g*) for each ROI where the mean and standard deviation (or standard error of mean) were reported. Data synthesis was conducted as a narrative report with tables used to summarize and demonstrate the patterns of alteration in magnetic susceptibility across the reviewed studies for each neurodegenerative disease.

## Results

### Search Summary

**Figure 1:**
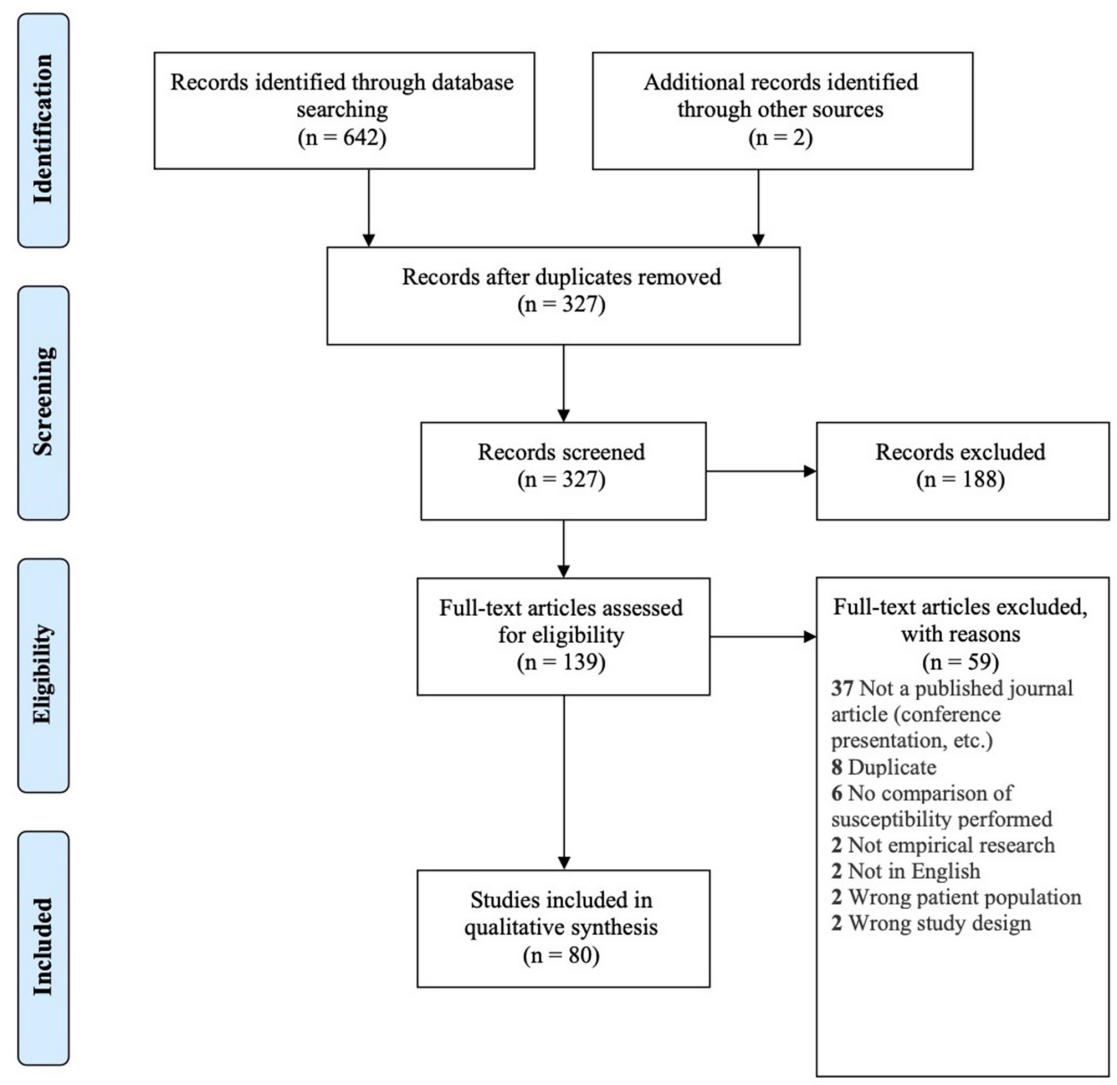
PRISMA flow diagram for inclusion of records (Moher et al., 2009).

A total of 642 records were found in our search across the four different databases. After removal of 315 duplicates, the remaining 327 studies were screened for eligibility, of which 80 were included in our review (see Figure 1 for PRISMA flow diagram).

The neurodegenerative diseases investigated in these studies included: AD, PD, HD, amyotrophic lateral sclerosis (ALS), Wilson's disease (WD) and rare genetic neurodegenerative diseases including Friedreich's ataxia (FRDA), multiple system atrophy (MSA), myotonic dystrophy (DM), spinocerebellar ataxia (SCA), progressive supranuclear palsy (PSP), Fabry disease, and syndrome of neurodegeneration with brain iron accumulation (NBIA). Table 1 shows the number of studies found for each neurodegenerative disease.

**Table 1:** Number of records included in this review for each neurodegenerative disease.

**Neurodegenerative disease**	**Number of studies included**
Alzheimer's disease	13
Parkinsonian diseases	43
Amyotrophic lateral sclerosis	8
Wilson's disease	4
Huntington's disease	3
Friedreich's ataxia	2
Spinocerebellar ataxia	2
Fabry disease	1
Myotonic dystrophy	1
Pantothenate-kinase-associated neurodegeneration	2
Mitochondrial membrane protein-associated neurodegeneration	1

In the following sections, we will present the findings of the review for each neurodegenerative disease structured into the following subheadings: (1) changes in tissue magnetic susceptibility when compared to healthy controls; (2) correlations between QSM findings and clinical features or other pathologic biomarkers; and (3) accuracy of QSM in differentiation of patients from healthy individuals. Reported changes of susceptibility in the brain regions examined by each study and the calculated effect size for the inter-group differences are summarized in color-coded Tables 2A–J. Detailed characteristics, main findings and quality assessment of studies are provided in the Supplementary Tables 1A–H. A summary of susceptibility changes across all neurodegenerative diseases reviewed in this paper are presented in Table 3.

**Table 2:**
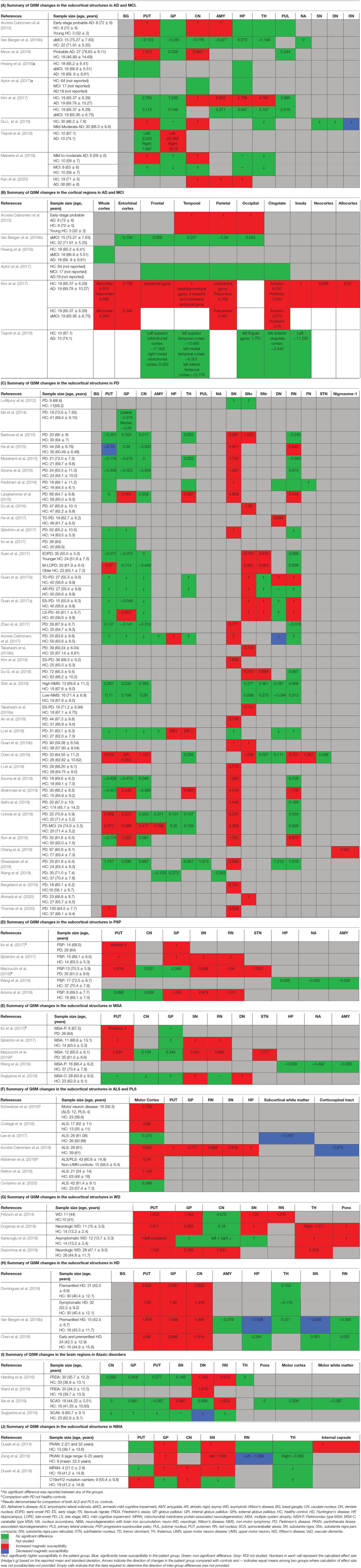
Color-coded tables for magnetic susceptibility changes in the regions of interest in each neurodegenerative disease.

**Table 3:** Summary of the methods used for QSM processing and referencing in the reviewed studies.

**Scanner strength**	**Field strength**	1.5T			1.5T/3T		3T		7T		9.4T					
	**Number of studies**	2			2		66		9		1					
Phase unwrapping	Method	Laplacian-based		Best path	Magnitude-guided spatial unwrapping		FSL prelude		Quality-guided		One-dimensional temporal unwrapping					Not reported
	Number of studies	30		1	3		2		3		1					40
Background field removal	Method	SHARP	RE-SHARP		V-SHARP	TSVD-SHARP		HARPERELLA		PDF						Not reported
	Number of studies	10	3		26	1		1		9		JEDI	1	MSDI	2	27
Dipole inversion	Method	LSQR	iLSQR	SFCR	HEIDI	MEDI	SDI	MUDICK	STAR-QSM	TKD	Other					Not reported
	Number of studies	4	19	1	3	13	2	1	4	2	4					24
Reference regions	Tissue type	CSF		White matter		Whole brain			Calcarine sulcus		Not referenced					Not reported
	Number of studies	18		19		7			1		2					33

*CSF, cerebrospinal fluid; HARPERELLA, harmonic phase removal with the Laplacian operator; HEIDI, homogeneity enabled incremental dipole inversion; iLSQR, iterative LSQR; JEDI, joint background-field removal and segmentation-enhanced dipole inversion; LSQR, sparse linear equation and least-squares; MEDI, morphology enabled dipole inversion; MSDI, multi-scale dipole inversion; MUDICK, multiple dipole-inversion combination with k-space segmentation; PDF, projection onto dipole fields; RE-SHARP, regularization-enabled SHARP; SDI, superfast dipole inversion; SFCR, structural feature-based collaborative reconstruction algorithm; SHARP, Sophisticated Harmonic Artifact Reduction for Phase; STAR-QSM, streaking artifact reduction for QSM; TKD, truncated k-space division; TSVD-SHARP, truncated singular value decomposition SHARP; V-SHARP, variable-radius SHARP*.

As shown in Table 1, there are very few QSM studies published in most neurodegenerative diseases. To adhere to the reporting standards for systematic reviews, we have included and presented the findings of all existing studies. However, to improve the readability of this paper, for the rare neurodegenerative diseases where fewer than three studies have been published (FRDA, SCA, Fabry disease, DM, PKAN, and MPAN), their relevant subheadings in the results and discussion sections have been presented in Supplementary Material 3 instead of the main text. Although, in the same way as the other disorders, their findings are presented in the Supplementary Tables 1F–H, 2, and the color coded tables (Tables 2I,J, 3).

### Alzheimer's Disease and Mild Cognitive Impairment (MCI)

#### Magnetic Susceptibility Changes in Alzheimer's Disease and Mild Cognitive Impairment in Comparison With Healthy Individuals

This review included 10 studies that used QSM to evaluate brain iron changes in AD, five studies that included a group of MCI subjects (with or without an AD group), and two studies that investigated the association of QSM and Aβ or APOE4 gene in a healthy population (see Supplementary Table 1A).

The comparison of magnetic susceptibility in the subcortical structures among people with AD and healthy individuals revealed inconsistent findings. The most consistent evidence for increased susceptibility was observed in the amygdala, caudate nucleus (CN), and putamen. All three studies that investigated the amygdala reported increased susceptibility in both mild (Acosta-Cabronero et al., 2013) and moderate (Kim et al., 2017) stages of AD (Kan et al., 2020). In people with amnestic MCI (aMCI), the amygdala did not show any significant difference compared to healthy individuals (Van Bergen et al., 2016b). In the CN, six out of seven studies reported higher susceptibility in AD. This increase was detected in both mild and moderate stages of AD (Acosta-Cabronero et al., 2013; Moon et al., 2016; Kim et al., 2017; Du L. et al., 2018; Meineke et al., 2018; Kan et al., 2020) but not in MCI (Hwang et al., 2016; Van Bergen et al., 2016b; Ayton et al., 2017; Meineke et al., 2018). Only a few studies reported the mean susceptibility values in the investigated ROIs to enable the estimation of the effect size of differences between groups (Table 2A).

Among seven studies that investigated the putamen, four reported higher susceptibility in both mild and moderate AD (Acosta-Cabronero et al., 2013; Moon et al., 2016; Du L. et al., 2018; Meineke et al., 2018), while in two studies on mild to moderate and moderate AD stages, no changes were found (Hwang et al., 2016; Kim et al., 2017). One study did not provide a description of disease stage in the study population (Ayton et al., 2017). In studies of patients with MCI, none detected any change in susceptibility in the putamen (Hwang et al., 2016; Van Bergen et al., 2016b; Ayton et al., 2017; Kim et al., 2017; Meineke et al., 2018).

Among other studied regions, limited evidence for increased susceptibility was provided in moderate AD in the hippocampus (two out of six studies) (Kim et al., 2017; Kan et al., 2020), in mild-moderate AD in the globus pallidus (GP) (one out of six studies) (Tiepolt et al., 2018) and in moderate AD in the thalamus (one out of four studies) (Kim et al., 2017) (Table 2A).

Cortical gray matter was examined in five studies. Patients with early-stage AD showed increased susceptibility in widespread regions over the temporal, parietal and occipital cortices (Acosta-Cabronero et al., 2013). Further, in studies of moderate AD, clusters of increased susceptibility were distributed across the cortex including the frontal, parietal, temporal, limbic, and insular lobes (Supplementary Table 1A) (Kim et al., 2017). Other studies, however, did not report any difference of mean susceptibility in the cortex between patients with mild or moderate AD and healthy individuals (Hwang et al., 2016; Ayton et al., 2017; Tiepolt et al., 2018). Among four studies that investigated QSM in the cortex in individuals with MCI; one reported increased susceptibility in the precuneus, allocortex, and anterior and posterior cingulate gyrus (Kim et al., 2017), while the others did not detect any difference between the MCI and healthy groups (Hwang et al., 2016; Van Bergen et al., 2016b; Ayton et al., 2017) (Table 2B).

#### Correlation of QSM Findings in AD and MCI With Clinical Features and Other Pathologic Biomarkers

Few studies examined the relationship between QSM changes and severity of cognitive deficits in AD. An association of magnetic susceptibility with mini-mental state examination (MMSE) and Montreal cognitive assessment (MoCA) in patients with AD was reported in the left CN (Du L. et al., 2018) and GP (Tiepolt et al., 2018). However, such correlations were not replicated in the study by Moon et al. (2016). In a longitudinal study combining Aβ positron emission tomography (PET) and QSM in AD, MCI, and cognitively normal individuals, Ayton et al. (2017) followed the study population by serial neuropsychiatric assessments and neuroimaging scans over 6 years. Interestingly, in both Aβ-positive and -negative subjects, magnetic susceptibility within certain ROIs was associated with deficits in specific cognitive domains. In the Aβ-negative group, higher QSM in the frontal lobe and CN was associated with subtle deterioration in language function. In the Aβ-positive individuals (MCI and AD), magnetic susceptibility of the hippocampus correlated with decline in episodic memory, attention and executive function in serial assessments, while susceptibility values in the temporal and frontal lobes predicted decline in language functions.

In a limited number of studies, the correlation of Aβ deposition and increased magnetic susceptibility in QSM was investigated in AD. Ayton et al. (2017) reported a significant correlation of Aβ-PET signal and QSM in the frontal, parietal, and occipital cortices but not in the parietal lobe, hippocampus, CN, or cingulate gyrus. On the other hand, in the study by Tiepolt et al. (2018), where authors examined the correlation of magnetic susceptibility and Aβ-PET only in the GP, no such correlation was detected. In a recent study, ultra-high field (9.4 and 14.1T) QSM at a resolution of 37 μm isotropic voxels in the frontal cortex of a post-mortem brain from a patient with AD showed that the pattern of increased susceptibility in the cortical layers strongly matched the Aβ depositions in histochemical staining (Tuzzi et al., 2020). In individuals with MCI, increased susceptibility significantly correlated with Aβ deposition in the cortical regions where altered functional coupling with the medial prefrontal cortex was shown in fMRI (Van Bergen et al., 2016b), while in healthy individuals, the significant correlation of magnetic susceptibility and Aβ PET signal was detected in clusters spread across the cortex and subcortical gray matter, with the strongest correlations in the GP, CN, and putamen (Van Bergen et al., 2018).

The association between APOE-e4 gene and QSM was investigated in two studies. Van Bergen et al. (2016b) studied a group of MCI subjects (*n* = 15) in comparison with healthy controls (*n* = 22). Forty percent of the MCI group and thirty-one precent of the control group were APOE-e4 carriers. While no differences of magnetic susceptibility in any of the ROIs existed between the MCI and healthy groups, in the MCI group, APOE-e4 carriers had significantly higher susceptibility in the CN as well as frontal, parietal, occipital, and temporal cortices than APOE-e4 negative subjects. In another study, Kagerer et al. (2020) reported no significant variation of susceptibility between APOE-e4 positive and APOE-e4 negative cognitively healthy adults. However, increased magnetic susceptibility and positive APOE-e4 gene status, were synergistically associated with increased default mode network activity. This relationship was most pronounced in the posterior cingulate cortex, precuneus, and lateral parietal cortex. In this study, the authors did not report any correlation with the cognitive control network, which would be relevant to the disorders of cognition.

#### Diagnostic Accuracy of QSM in Differentiation of Patients With AD From Healthy Individuals

A number of studies used the Receiver Operating Characteristic (ROC)-curve analysis to evaluate the diagnostic accuracy of QSM in differentiation of people with AD and MCI from healthy subjects. In AD vs. healthy controls, high sensitivity, and specificity was observed for magnetic susceptibility of the GP (90 and 100%, respectively) (Tiepolt et al., 2018), while the CN showed lower accuracy (sensitivity and specificity = 61.67%) (Du L. et al., 2018).

Kim et al. (2017) examined both AD and MCI in comparison with healthy controls. The area under curve (AUC) was 0.850, 0.831, and 0.803 for susceptibility of the precuneus, amygdala, and hippocampus, respectively, showing good accuracy in differentiation of AD from healthy groups. Furthermore, in differentiation of people with MCI from healthy individuals, susceptibility in the hippocampus, thalamus, precuneus, and various cortical areas showed moderate accuracy (AUC: 0.692–0.759). In the comparison between MCI and healthy controls by Hwang et al. (2016), the mean susceptibility of the white matter yielded a sensitivity of 55.56% and specificity of 94.44%.

### Parkinsonian Diseases

Forty-three studies using QSM in the investigation of brain iron in PD and other parkinsonian diseases, such as MSA, PSP, and corticobasal degeneration were included in this review.

#### Magnetic Susceptibility Changes in Parkinsonian Diseases in Comparison With Healthy Individuals

**Figure 2:**
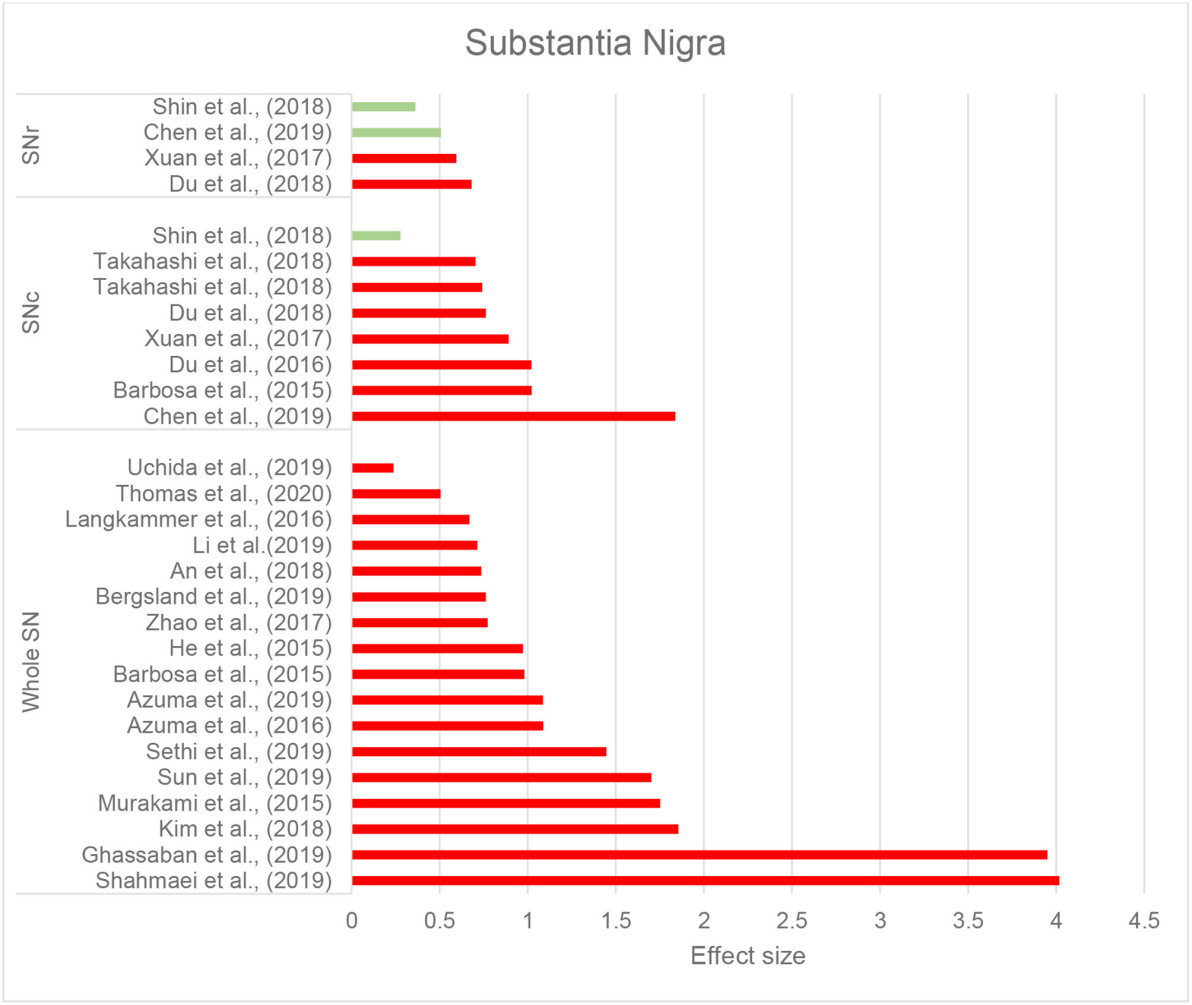
Parkinson's disease: Effect sizes for the intergroup differences of magnetic susceptibility in the SN, SNr, and SNc between PD and healthy groups. Red bars indicate significantly higher susceptibility in the patient group, while green color shows a non-significant difference.

**Figure 3:**
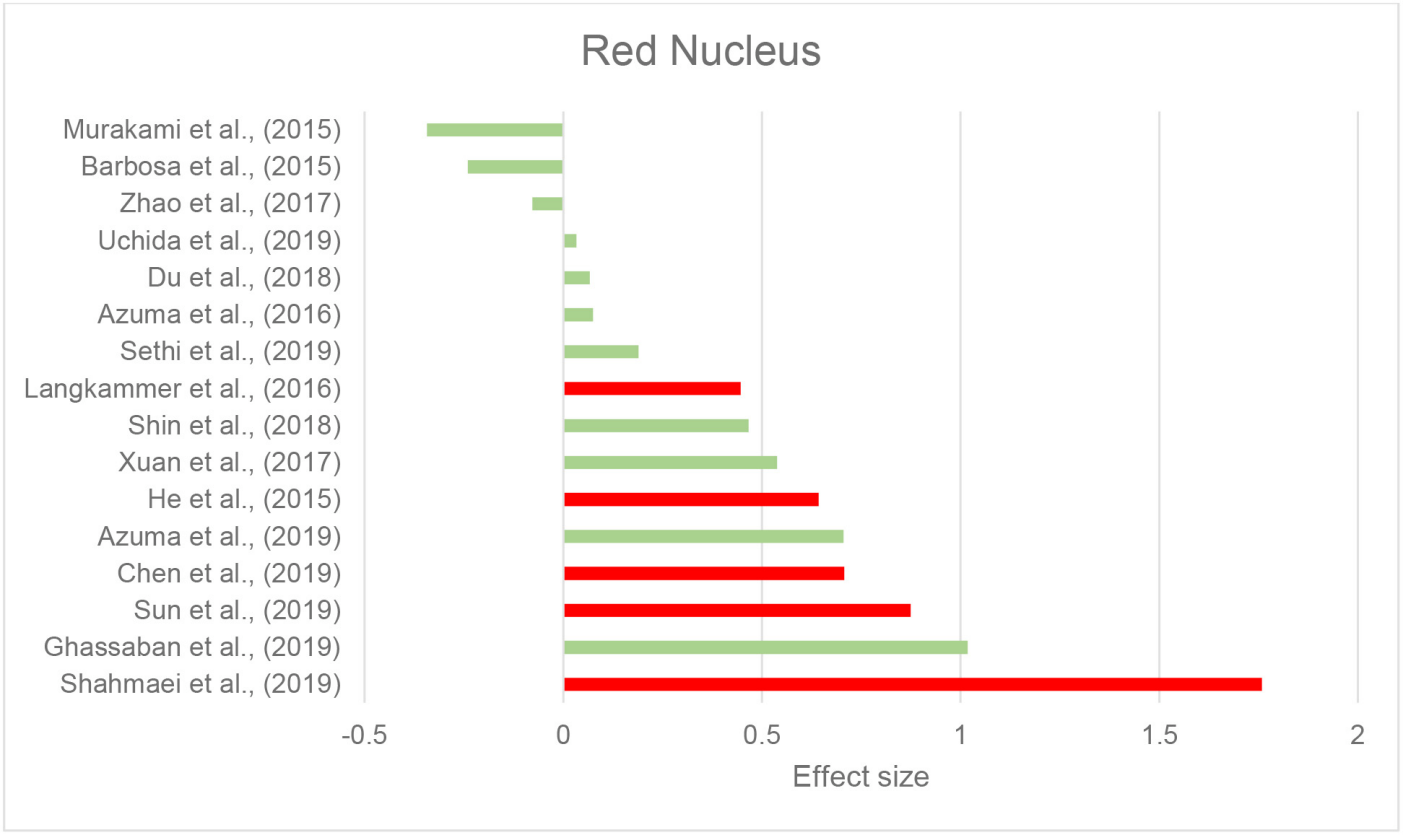
Parkinson's disease: Effect sizes for the intergroup differences of magnetic susceptibility in the RN between PD and healthy groups. Red bars indicate significantly higher susceptibility in the patient group, while green color shows a non-significant difference.

Thirty-seven studies reported the measurement and comparison of magnetic susceptibility in the subcortical gray matter structures among the PD and control groups. Of this, 33 investigated the SN or its subregions [SNc and substantia nigra pars reticulata (SNr)], with 30 (90.9%) reporting significantly increased magnetic susceptibility in PD (Table 2C). Investigated in one study, magnetic susceptibility in the SN was also increased in idiopathic REM sleep behavior disorder which is considered as a prodromal phase for synucleinopathies with high rates of conversion to PD (Sun et al., 2019). The next most investigated region was the red nucleus (RN) (22 studies), where seven studies (31.8%) reported increased susceptibility in PD (Table 2C). Figures 2, 3 demonstrate the effect sizes for susceptibility differences in the SN and RN respectively, in patients with PD compared to healthy individuals.

The alterations in susceptibility were less consistent in the basal ganglia in PD patients. Only four of 22 studies (18.1%) of the putamen showed a significant increase in susceptibility while one study reported significantly decreased susceptibility in the putamen (He et al., 2015). Six of 21 studies (28.6%) investigating the GP reported increased susceptibility and only one of 18 studies (5.5%) reported significantly higher susceptibility in the CN. It is worth mentioning that, while not statistically significant, a trend toward lower susceptibility in the basal ganglia was reported in a noticeable proportion of the studies (Putamen: 8 out of 22, GP: 8 out of 21, and CN: 9 out of 18) (Table 2C).

In the hippocampus, significantly unilateral (Li et al., 2018) or bilateral (Acosta-Cabronero et al., 2017) higher susceptibility was reported in two out of four studies. In the thalamus, and cerebellar dentate nucleus (DN), most of the studies reported no significant changes in regional magnetic susceptibility. These findings are summarized in Table 2C and Supplementary Table 1B.

In one of the first studies to investigate QSM in cortical gray matter in PD, Acosta-Cabronero et al. (2017) reported increased susceptibility in widespread regions of the cortex including the lateral occipital, posterior parietal, rostral middle prefrontal, and middle temporal cortex. In a further update, the same group reported increased susceptibility in prefrontal and right insular cortices in PD (Thomas et al., 2020). In the study by Uchida et al. (2019), higher susceptibility was observed in the cuneus, precuneus, fusiform gyrus, insula and cerebellum in PD patients. None of these studies found any brain region with lower susceptibility in patients with PD. On the other hand, the mean susceptibility value of the whole cortex did not show any difference between patients with PD and healthy controls in the study by Chen et al. (2019). Surprisingly, Lewy-body dementia, which is characterized by cortical involvement by the same pathology as PD (α-synuclein), has not been examined in QSM studies.

In one study investigating white matter QSM in PD, Guan et al. (2019a) reported alterations in widespread areas in the frontal, temporal, and parietal lobes (details in Supplementary Table 1B). In the inferior longitudinal fasciculus, there were increases in both magnetic susceptibility and radial diffusivity measured by diffusion tensor imaging (DTI).

In six studies, QSM was compared between controls and PD or other parkinsonian syndromes, such as PSP and MSA (Tables 2D,E). Compared to healthy controls, susceptibility was increased in the RN, SN, and GP in PSP (Sjöström et al., 2017; Azuma et al., 2019), in the RN, putamen, and SN, in MSA (Sjöström et al., 2017), and in the DN and SN in cerebellar type of MSA (MSA-C) (Sugiyama et al., 2019). Compared to PD, magnetic susceptibility was higher in the SN, subthalamic nucleus (STN), putamen, and RN in both patients with MSA and PSP, while in PSP, higher susceptibility was further detected in the GP (Ito et al., 2017; Sjöström et al., 2017; Azuma et al., 2019; Mazzucchi et al., 2019). In comparison with PD, patients with parkinsonism dominant MSA (MSA-P) showed higher susceptibility in the posterior putamen (Ito et al., 2017), lateral SN, STN, and RN (Mazzucchi et al., 2019) (Supplementary Table 1B).

Studies comparing PSP and MSA reported higher susceptibility of the GP, RN, and the medial part of the SN in PSP (Sjöström et al., 2017; Mazzucchi et al., 2019). The only study that compared limbic structures among PD, MSA, and PSP using QSM, reported no susceptibility differences in the hippocampus, amygdala, and nucleus accumbens (Wang et al., 2019). Nevertheless, it should be noted that in some of the studies on MSA, PSP, and PD, study groups were not matched based on age and disease characteristics. In the study by Mazzucchi et al. (2019), patients with PSP were older than the MSA and PD groups. In the study by Ito et al. (2017) patients with PD were at lower disease stages compared to the other groups. Also in the studies by Mazzucchi et al. (2019) and Wang et al. (2019), the PD group had lower clinical severity scores compared to the other groups.

#### Correlation of QSM Findings in PD With Clinical Features and Other Pathologic Biomarkers

In addition to the comparison of QSM in various brain regions among PD and healthy individuals, studies have also evaluated its correlation with duration and severity of symptoms, disease stage, and clinical features of PD. Magnetic susceptibility of the SN (He et al., 2015) and SNc (Du et al., 2016) were shown to positively correlate with disease duration, however, such a correlation has not been agreed upon by all studies (Ghassaban et al., 2019). Additionally, this correlation was not found in any of the other subcortical structures including the GP, putamen, CN, thalamus, RN, and cerebellar DN (Barbosa et al., 2015; Langkammer et al., 2016; Shin et al., 2018; Sun et al., 2019).

In the PD studies included, disease progression stage was generally assessed by Hoehn and Yahr (H&Y) scale and clinical severity of symptoms were evaluated by Unified Parkinson's Disease Rating Scale (UPDRS). H&Y staging evaluates the overall clinical progression of motor impairments in PD on a scale of 1–5, with a score of 1 indicating limited unilateral involvement and 5 indicating a wheelchair-bound or bedridden state (Goetz et al., 2004). UPDRS is a PD severity assessment scale, comprised of three parts: UPDRS-I evaluates mental status and cognition; UPDRS-II assesses the impairment of daily activities; and UPDRS-III evaluates the severity of motor dysfunction (Movement Disorder Society Task Force on Rating Scales for Parkinson's Disease, 2003).

The majority of evidence indicates a significant association of susceptibility in the SN and its subfields with H&Y illness stage (Langkammer et al., 2016; Guan et al., 2017a,b; Xuan et al., 2017; An et al., 2018; Chen et al., 2019; Shahmaei et al., 2019). A simlar association was found in the RN and GP in some (Guan et al., 2017a; Chen et al., 2019; Shahmaei et al., 2019), but not all studies (Shin et al., 2018; Sun et al., 2019).

The correlation of UPDRS sub-scores have been evaluated by a number of studies, showing inconsistent findings. While some studies suggested a correlation of UPDRS scores with susceptibility in subcortical structures, such as CN, GP, putamen, and SN (He et al., 2015; Du et al., 2016; Langkammer et al., 2016; Guan et al., 2017b; Xuan et al., 2017; An et al., 2018; Du G. et al., 2018; Mazzucchi et al., 2019; Uchida et al., 2019; Thomas et al., 2020), others did not report such findings (Du et al., 2016; Acosta-Cabronero et al., 2017; Zhao et al., 2017; Shin et al., 2018; Bergsland et al., 2019; Ghassaban et al., 2019; Sun et al., 2019) (Supplementary Table 1B).

##### Tremor Dominant vs. Akinetic-Rigid PD

Tremor-dominant and akinetic-rigid clinical presentations of PD were found to have distinct QSM patterns in specific regions of the brain. While in the SN, magnetic susceptibility did not differ among tremor-dominant and akinetic-rigid groups, the cerebellar DN and RN showed higher susceptibility in tremor-dominant PD, which positively correlated with tremor severity (Guan et al., 2017b; He et al., 2017; Mazzucchi et al., 2019). Additionally, the patients with akinetic-rigid PD showed higher susceptibility in the CN compared to those with tremor-dominant disease (Guan et al., 2017b).

##### Cognitive Impairment in PD

In patients with PD, cognitive impairment was significantly correlated with higher susceptibility in the hippocampus, rostral CN, thalamus, amygdala, and right putamen (Li et al., 2018; Uchida et al., 2019; Thomas et al., 2020) but not in the SN and its subregions, midbrain, and DN (He et al., 2017; Du G. et al., 2018; Ahmadi et al., 2020). Cortical regions where increased susceptibility correlated with lower MoCA scores include the basal forebrain, caudal regions of ventromedial prefrontal cortex, right insular cortex (Thomas et al., 2020), cuneus, and fusiform gyrus (Uchida et al., 2019).

Further, compared to patients with PD who had normal cognition, those with MCI showed higher susceptibility in the head of CN, entorhinal cortex, parahippocampal gyrus, amygdala, and precuneus (Uchida et al., 2019). In another study, Li et al. (2018) reported higher susceptibility in the left hippocampus in patients with PD with dementia, compared to cognitively normal PD patients.

##### Olfaction in PD

Hyposmia is a common non-motor symptom in PD which occurs in up to 90% of patients (Pantelis and Brewer, 2006; Xiao et al., 2014). Two studies compared QSM among PD patients with and without olfactory deficits. Uchida et al. (2019) reported that higher susceptibility in the amygdala, cuneus and fusiform gyrus was associated with olfactory deficits. However, in the study by Hwang et al. (2019), differences were only observed in the thalamus, with significantly higher susceptibility in the left thalamus and lower susceptibility in the right thalamus (showing a more heterogeneous distribution of susceptibility) in patients with hyposmia.

#### Diagnostic Accuracy of QSM in Parkinsonian Diseases

In nine studies, ROC analysis was used to evaluate the accuracy of QSM in differentiating parkinsonian disease groups. QSM in the SN was able to differentiate patients with PD from healthy individuals with an accuracy of 68–88.7% (Azuma et al., 2016; Zhao et al., 2017; Takahashi et al., 2018a,b; Li et al., 2019), with the highest accuracy in the posterior part of SN (Azuma et al., 2016). Magnetic susceptibility in the DN could differentiate patients with MSA-C from healthy individuals with an accuracy of 83.4% (Sugiyama et al., 2019), while magnetic susceptibility in the posterior putamen (AUC = 0.91) (Ito et al., 2017), RN (AUC = 0.86) (Sjöström et al., 2017), and STN (AUC = 0.808) differentiated MSA from PD (Mazzucchi et al., 2019). The highest accuracy for differentiation of PSP from PD has been reported for susceptibility in the GP (AUC = 0.903) (Azuma et al., 2019) and RN (AUC = 0.97) (Sjöström et al., 2017), while the accuracy of QSM in differentiating between MSA and PSP, was 0.826 in the RN, 0.785 in the medial part of the SN (Mazzucchi et al., 2019), and 0.73 in the GP (Sjöström et al., 2017).

### Amyotrophic Lateral Sclerosis (ALS) and Primary Lateral Sclerosis (PLS)

#### Magnetic Susceptibility Changes in ALS and PLS in Comparison With Healthy Individuals

**Figure 4:**
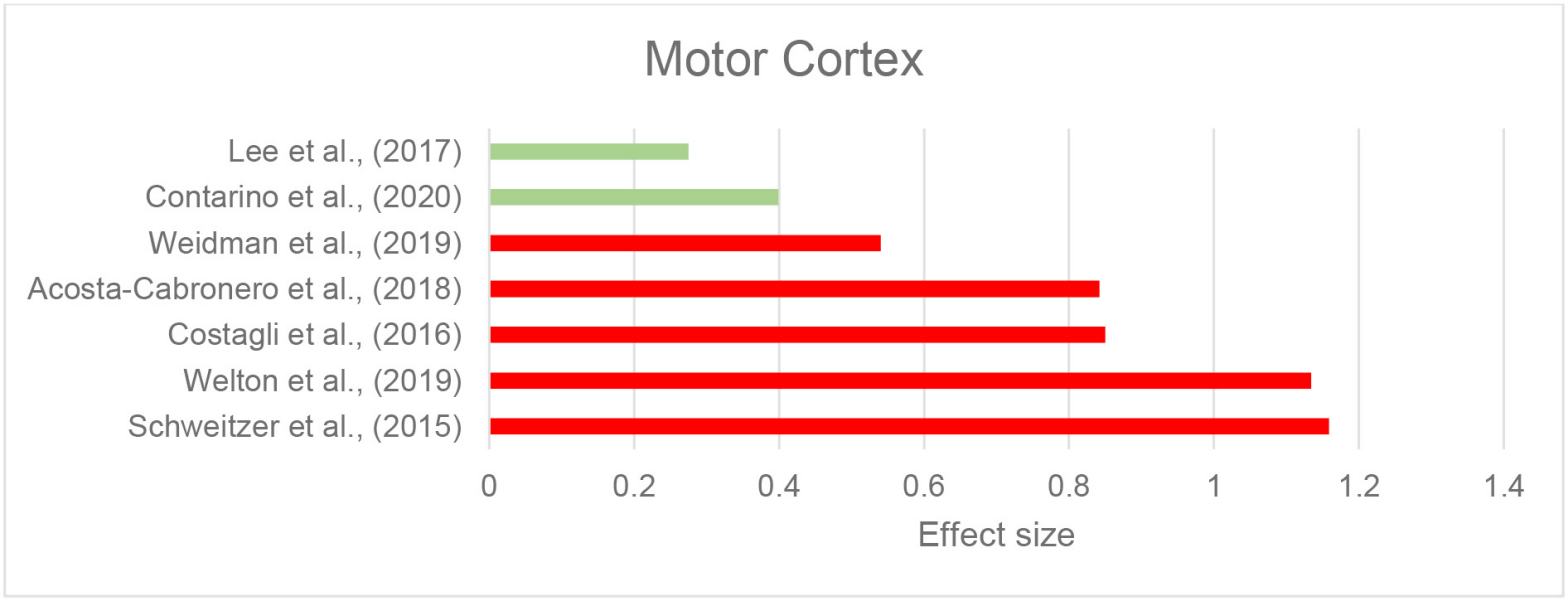
Amyotrophic lateral sclerosis: Effect sizes for the intergroup differences of magnetic susceptibility in the motor cortex between ALS and control groups. Red bars indicate significantly higher susceptibility in the patient group, while green color shows a non-significant difference.

We identified eight records investigating brain iron in patients with ALS using QSM, in which the motor cortex was the primary focus. The majority of studies reported higher susceptibility within the motor cortex compared to the control groups (Acosta-Cabronero et al., 2018; Welton et al., 2019) (Figure 4 and Table 2F). Costagli et al. (2016) reported focally increased susceptibility in the deep layers of the hand and foot motor cortices corresponding to the most affected limb, compared to healthy controls. Such increase was primarily observed in ALS patients who had evident T2^*^ hypoinstensities in the deep cortical layers and was not found in those without the T2^*^ hypoinstensities. In the study by Contarino et al. (2020), the entire motor cortex including the precentral gyrus and paracentral lobule was examined as one ROI. The results revealed a close-to-significant trend of higher mean susceptibility, and a significantly higher skewness and standard deviation of susceptibility values across the motor cortex in ALS patients, which indicates increased heterogeneity of susceptibility distribution. One study did not observe any susceptibility difference in the motor cortex of patients with ALS compared with controls, but reported a significantly larger susceptibility variation from the motor cortex to the adjacent subcortical white matter in patients with ALS compared to healthy individuals (Lee et al., 2017) (Supplementary Table 1C).

Comparison of individuals with ALS and PLS with healthy controls revealed higher maximum (Weidman et al., 2019) and mean (Schweitzer et al., 2015) susceptibility in the motor cortex in the patients with ALS or PLS but no difference existed between patients with ALS and those with PLS (Schweitzer et al., 2015). In the only study to investigate the subcortical structures and whole brain susceptibility changes in ALS, Acosta-Cabronero et al. (2018) reported a markedly increased susceptibility in the motor cortex, pars opercularis of the prefrontal cortex, premotor medial areas, and the primary somatosensory fields, SN, RN, GP, putamen, and hippocampus in patients with ALS. Tissue magnetic susceptibility was significantly lower in the corticospinal tract (CST), which positively correlated with fractional anisotropy and negatively correlated with mean diffusivity and radial diffusivity of the tract measured by DTI.

#### Correlation of QSM Findings in ALS With Clinical Features and Other Biomarkers

The correlation of QSM findings of altered susceptibility with disease severity and duration was investigated in four studies, but the results were inconsistent. Two studies reported that susceptibility was not associated with disease severity (Acosta-Cabronero et al., 2018; Welton et al., 2019) or duration (Welton et al., 2019; Contarino et al., 2020). In contrast, two studies reported that disease severity was associated with the median susceptibility of the motor cortex (Contarino et al., 2020) and mean and maximum susceptibility of the left motor cortex (Lee et al., 2017).

In a recent study, the orofacial region of the primary motor cortex was investigated in relation to bulbar onset of ALS presentation. Magnetic susceptibility was significantly higher in those with marked T2^*^ hypointensity in the deep layer of orofacial region of the motor cortex, which was predominantly observed in patients with bulbar-onset disease (Donatelli et al., 2019).

#### Diagnostic Accuracy of QSM in Differentiating Patients With ALS From Healthy Individuals

The ROC curve analysis of QSM in the motor cortex showed an AUC of 0.632 for the maximum (Lee et al., 2017) and 0.70 for the mean susceptibility of the motor cortex relative to the adjacent white matter (sensitivity: 74%, specificity: 54.8%) for the differentiation of ALS from controls. In the study by Schweitzer et al. (2015), susceptibility of the motor cortex showed high accuracy for the differentiation of patients with ALS and PLS from healthy controls (AUC: 0.88, sensitivity: 87.5%, specificity: 87%).

### Wilson's Disease

There were four articles included in this review that described the use of QSM in WD, two studies in pediatric patients with WD and two studies on adult patients with WD, with comparisons to age-matched healthy individuals (Table 2G and Supplementary Table 1D).

Doganay et al. (2018) found increased susceptibility in the putamen, CN, SN, thalamus, and pons in 11 pediatric patients (mean age = 15 years) with neurological-WD compared to healthy controls, while T1 and T2 images did not show any differences among the groups. The same research group investigated brain QSM changes in 12 asymptomatic children (mean age 13.3 years) with a positive WD mutation. QSM evaluation of the same ROIs as the previous study revealed a similar pattern of higher susceptibility except for GP and SN (Saracoglu et al., 2018).

In the first QSM study in adult WD, Fritzsch et al. (2014) used a 7T MRI scanner to investigate brain changes in 11 adult patients with WD (six with neurologic WD, and five with hepatic WD) with a mean age of 44, and 10 age-matched healthy controls. The results showed a differential pattern of increased susceptibility in the subcortical structures among patients with neurologic and hepatic WD compared to controls. In both groups of patients with WD, the SN and right GP showed higher susceptibility. Susceptibility was further found to be increased in the left GP and putamen in neurologic WD group, and in the RN in patients with hepatic WD (Fritzsch et al., 2014).

**Figure 5:**
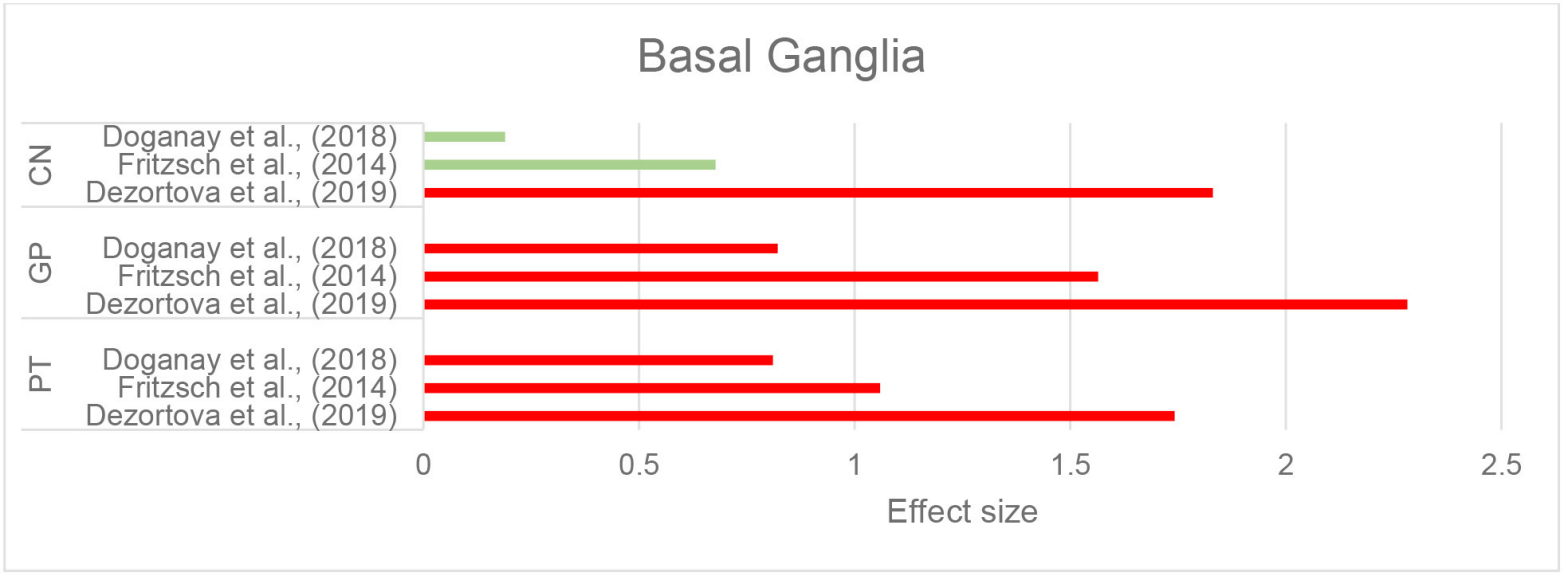
Wilson's disease: Effect sizes for the intergroup differences of magnetic susceptibility in the basal ganglia between WD and healthy groups. Red bars indicate significantly higher susceptibility in the patient group, while green color shows a non-significant difference.

Dezortova et al. (2019) used a 3T MRI scanner to study a larger sample of participants with WD (28 with neurologic-WD and 10 with mild hepatic-WD) and comparisons were made with 26 healthy controls. In addition to increased susceptibility in the putamen and GP in neurologic WD, similar to the findings of Fritzsch et al. (2014), the CN and thalamus also showed this increase. Of note, this pattern was only observed in the neurologic WD group, while patients with hepatic WD showed no significant differences in any of these regions compared to healthy controls (Dezortova et al., 2019). Figure 5 demonstrates the effect sizes for increased magnetic susceptibility in the basal ganglia in patients with WD compared to healthy individuals.

Correlation of clinical assessments with QSM and the accuracy of QSM in the differentiation of patients with WD from healthy individuals have not been examined in these studies.

### Huntington's Disease

#### Magnetic Susceptibility Changes in HD in Comparison With Healthy Individuals

**Figure 6:**
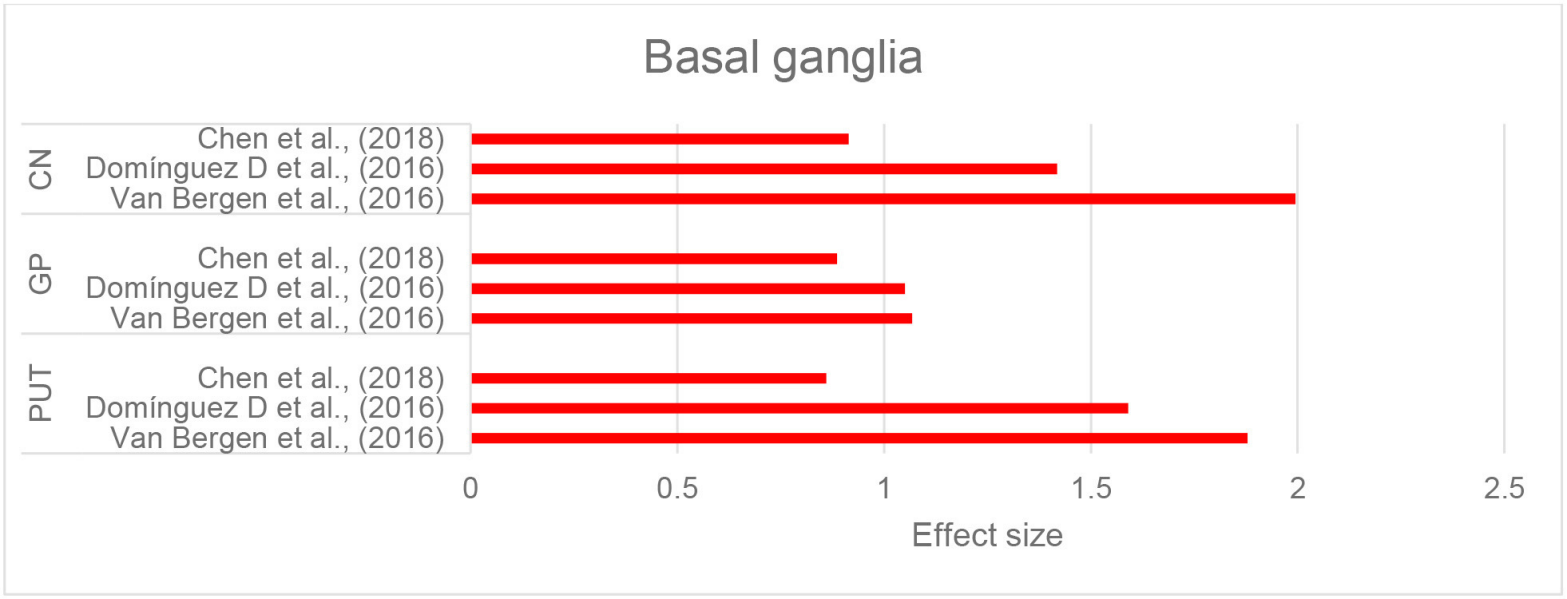
Huntington's disease: Effect sizes for the intergroup differences of magnetic susceptibility in the basal ganglia between HD and control groups. Red bars indicate significantly higher susceptibility in the patient group.

Three studies that investigated brain iron distribution in HD using QSM were included in this review. These studies consistently reported higher susceptibility in the CN, GP, and putamen in both asymptomatic carriers of the HD gene mutation and in patients with HD compared to healthy controls (Domínguez et al., 2016; Van Bergen et al., 2016a; Chen et al., 2018) (Supplementary Table 1E, Figure 6, and Table 2H). Increased susceptibility within these regions also significantly correlated with their atrophy (Van Bergen et al., 2016a; Chen et al., 2018).

In the thalamus and RN, QSM did not show any alterations in HD compared to healthy controls (Domínguez et al., 2016; Van Bergen et al., 2016a; Chen et al., 2018). In one study investigating preclinical carriers of HD genotype, lower susceptibility in the SN and hippocampus was reported compared to healthy individuals (Van Bergen et al., 2016a), however, such a finding was not observed in another study (Chen et al., 2018).

#### Correlation of QSM Findings in HD With Clinical Features and Other Pathologic Biomarkers

Higher susceptibility values in the CN, putamen, and GP significantly correlated with composite scales of age and genetic burden of the disease (CAG-age product scaled score, see Supplementary Table 1E) (Domínguez et al., 2016; Van Bergen et al., 2016a; Chen et al., 2018). The CAG-age product scale score is calculated based on age and length of CAG trinucleotide repeats, and estimates the probability of disease onset within 5 years (Zhang et al., 2011). This increase in susceptibility, however, was not correlated with clinical motor symptoms and function, as measured by the Unified Huntington Disease Rating Scale or cognitive assessments by MoCA in any of the studies (Domínguez et al., 2016; Van Bergen et al., 2016a; Chen et al., 2018).

### Methodological Diversity of QSM Processing in Reviewed Studies

Among the studies included in this review, we observed a variety of MRI acquisition sequences, post-processing methods and reference regions used to produce QSM images. Table 3 provides an overview of this methodological diversity. A detailed description of MRI acquisition and QSM processing methods implemented in each study is brought in the Supplementary Table 2.

#### Scanner Field Strength

QSM studies were most frequently conducted using scanners with the magnetic field strength of 3T (66/80 studies) (Table 3), while a smaller number of studies were carried out using 1.5T, 7T, or 9.4T scanners (2, 9, and 1 studies, respectively). Sixty-seven studies employed multi-channel receiver coils.

#### Data Acquisition Parameters

All studies performed QSM on MRI scans from single head orientation, with the majority of studies using a gradient echo sequence with or without flow compensation. Fourteen studies reported single-echo acquisition, and fifty-four studies acquired multi-echo data with inter-echo spacing varying between 2 and 12 ms. The first echo times ranged between 2 and 53 ms (Supplementary Table 2).

The spatial resolution is an important factor that affects the accuracy of the magnetic susceptibility estimates. Images with smaller voxels have higher spatial resolution. Sixteen studies had a voxel-size equal to or smaller than 0.5 × 0.5 × 2 mm^3^, which is the recommended voxel-size to ensure reasonably accurate magnetic susceptibility estimates (Haacke et al., 2015). Sixty-nine studies had voxel-sizes >0.5 × 0.5 × 2 mm^3^ (Table 4).

**Table 4:**
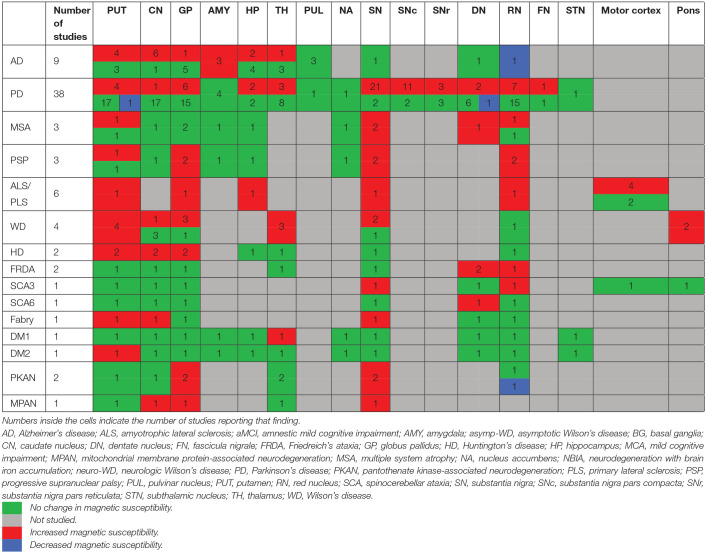
Summary of magnetic susceptibility changes in the brain regions across neurodegenerative diseases compared to healthy individuals.

#### Phase Unwrapping Methods

Laplacian-based phase unwrapping algorithms were employed by thirty studies to recover true phase of the MRI signal from the measured phase, which is wrapped between –π and π (Table 3). The Laplacian-based phase unwrapping techniques utilize spatial information in the Fourier space to correct for arbitrary 2π jumps present in the measured phase images (Schofield and Zhu, 2003; Deistung et al., 2017). Other phase unwrapping methods used in these studies include best path algorithm, FSL Prelude, magnitude- and quality-guided spatial unwrapping and temporal unwrapping. A detailed description of these methods can be found elsewhere (Schweser et al., 2017; Fortier and Levesque, 2018). Out of eighty studies included in this review, forty studies did not report the phase unwrapping method used to obtain QSM estimates.

#### Background Field Removal Algorithms

In order to identify local contributions to the magnetic susceptibility, 40 studies used a variant of Sophisticated Harmonic Artifact Reduction for Phase data (SHARP)-based background field removal techniques (Table 3). Such techniques eliminate the harmonic background magnetic field component using its spherical mean value property and reconstruct the non-harmonic local field component using a deconvolution operation on the filtered phase images (Schweser et al., 2011). SHARP-based methods are susceptible to cortical surface artifact, leading to limited accuracy in determining cortical magnetic susceptibility distribution. Advanced SHARP-based techniques use regularization (RE-SHARP) and variable high-pass filter sizes and regularization parameters (V-SHARP) to reduce surface and streaking artifacts in magnetic susceptibility maps (Schweser et al., 2017).

Nine studies used the projection onto dipole field (PDF) method, that uses the orthogonality between magnetic fields produced by dipoles outside and inside an ROI to eliminate background field susceptibility contributions (Liu et al., 2011).

#### Dipole Inversion

Twenty-eight studies employed a sparse linear equation and least-squares (LSQR)-based method to perform dipole inversion for the purpose of estimating magnetic susceptibility from local field shifts. The iterative LSQR (iLSQR) was the most frequently employed dipole inversion method. Thirteen studies used morphology enabled dipole inversion (MEDI) to supress artifacts using edge information from the magnitude images (Liu et al., 2011). Twenty-three studies did not report the dipole inversion method used for estimating magnetic susceptibility maps (Table 3).

#### Normalization of Magnetic Susceptibility Values

Magnetic susceptibility values were normalized using mean susceptibility values from ventricular CSF (18 studies), white matter (19 studies), or whole brain (seven studies), while two studies did not use referencing of the QSM values and thirty-three studies did not report their normalization process.

## Discussion

In the results section, we provided a report of the existing evidence supplied by QSM in neurodegenerative disorders. This section is structured in two main parts. In the first part, we will provide a synthesis of the QSM findings and their implications in each neurodegenerative disease. Similar to the results section, the discussion relating to the less studied neurodegenerative diseases (FRDA, SCA, Fabry disease, DM, PKAN, and MPAN) is moved to the Supplementary Material 3. In the second part, we provide a discussion of our overall current understanding, limitations of QSM and suggested future directions for QSM studies.

### The Implications of QSM Findings in Neurodegenerative Diseases

#### Alzheimer's Disease

Abnormal iron homeostasis has been hypothesized to play a role in the pathogenesis of AD, with evidence suggesting a bidirectional pathological interaction between iron and Aβ (Masaldan et al., 2019). Excess iron upregulates gene expression of amyloid precursor protein, shifts its physiologic non-amyloidogenic processing toward amyloidogenic cleavage that produces Aβ peptides, and contributes to the misfolding of Aβ peptides and production of insoluble Aβ plaques (Rogers et al., 2008). Aβ plaques in turn, absorb free iron, which enhances their neurotoxicity through production of reactive oxygen species and oxidative stress (Telling et al., 2017; Cheignon et al., 2018). Further, abnormal iron deposition has been detected in Aβ plaques in histologic evaluation of post-mortem brains from AD patients (Lovell et al., 1998), and APOE-e4 gene status, which is the most common predisposing genetic risk factor for AD, has been linked to increased iron in certain regions of the brain (Van Bergen et al., 2016b; Kagerer et al., 2020). These findings support the involvement of iron in the pathogenesis of AD.

Our review of studies using QSM in AD shows that there is an increased iron deposition in the CN and amygdala, in both mild and moderate stages of AD (Table 2B). The amygdala is one of the main regions involved in the pathologic processes in AD and undergoes significant atrophy in the early stages of AD. Atrophy of the amygdala is comparable to that of the hippocampus and correlates with cognitive deficits (Poulin et al., 2011). Although less investigated, the striatum shows evidence of Aβ deposition in the early stages of AD at even higher levels than the cortical regions, and volumetric assessments have revealed significant changes in the CN in AD. While some studies have reported reduced volumes, others found increased size of the CN in early AD (Tentolouris-Piperas et al., 2017; Persson et al., 2018). Although this inconsistency remains unexplained, these significant changes suggest the involvement of the CN in AD.

In the other subcortical and cortical regions, findings of susceptibility changes were inconsistent. The hippocampus, which is one of the key areas undergoing significant atrophy in AD (Blair et al., 2020), showed changes in susceptibility in only two out of six studies. Similarly, key cortical regions involved in AD, such as the medial temporal lobe, precuneus, and cingulate gyrus have been shown by a limited number of studies to have increased susceptibility, while others did not confirm these results. One reason for the inconsistent results in the hippocampus and cortical regions can be the inaccuracies in QSM measurements at the edges of the brain where environments with drastically difference susceptibilities meet. This limitation is further discussed in section Methodological Aspects of QSM. It is also important to note that the susceptibility measurements depend on the precise segmentation of an ROI. Inclusion of the surrounding tissues within a segmented ROI leads to mean susceptibility measurements that are less representative of the actual values. Experience shows that the segmentation of the hippocampus and amygdala are particularly challenging. This reduces the reliability of both positive and negative findings in these regions and warrants further evaluations with more accurate segmentation methods.

MCI is defined as the slight impairment of cognition often in the memory domain, but not sufficiently severe to be characterized as dementia. Individuals with MCI are at higher risk for AD but do not necessarily progress to AD (Kelley and Petersen, 2007). Amnestic MCI, however, is recognized as a precursor to AD (Morris et al., 2001) with conversion rates to AD estimated to be between 7.5 and 16.5% per year (Ward et al., 2013). The QSM findings in aMCI and MCI have been inconsistent. Based on the studies suggesting a colocalization of Aβ PET signal and increased susceptibility in QSM, it would be expected to detect changes in the brain regions where accumulation of Aβ plaques occur (Ayton et al., 2017). One possible explanation of the negative findings of QSM changes in MCI may be that the increased iron content in the regions with Aβ deposition does not reach the level that enables its detection by QSM. We did not find any longitudinal QSM studies in people with MCI to examine susceptibility changes over time and its association with progression to AD.

#### Parkinson's Disease

PD is the second most common neurodegenerative disease presenting with motor and cognitive impairment. The disease is characterized by degeneration and atrophy of the dopaminergic neurons in the SN along with intracellular α-synuclein deposition (Lewy bodies) (Poewe et al., 2017). In PD, nigral iron accumulation is suggested to result from dysregulation of iron transmembrane transporters, decreased ferritin, and impaired iron export mechanisms (Masaldan et al., 2019). Excess iron contributes to neurodegeneration by inducing the aggregation of alpha synuclein (Bharathi and Rao, 2007) and formation of Lewy bodies (Castellani et al., 2000), in addition to the pathways of oxidative toxicity, which are common across neurodegenerative diseases (Wang et al., 2017).

The centerpiece of pathology in PD is within the SN. Physiologically, SN is one of the iron-rich regions of the brain, storing large amounts of iron required for dopamine synthesis, mainly bound to neuromelanin (Sulzer et al., 2018). Abundance of iron in the SN enables highly accurate visualization of this region required for deep brain stimulation surgeries, which has resulted in a large number of published studies examining QSM in PD. These studies have shown consistent evidence of higher susceptibility in the SN in patients with PD compared to healthy individuals, suggestive of increased iron content. In other subcortical structures including the basal ganglia, RN, thalamus, and hippocampus, evidence for increased susceptibility has been inconsistent, with the majority of the studies finding no changes in PD.

Although susceptibility in the SN was increased in most studies, there have been inconsistent findings of its association with disease duration, stage and severity of clinical symptoms. This may relate to important confounders, such as medication. For example, in some studies, clinical assessments have been performed in the “on-state” (with dopaminergic agonist treatment) while in others, assessments were in the “off-state” (no dopaminergic treatment). Further, since more advanced stages of the disease inherently interfere with the ability of patients to participate in studies due to excessive movements throughout the MRI acquisition and other comorbidities, the populations of PD patients in studies are predominantly limited to earlier stages of the disease. It is possible that the selective inclusion of individuals who are at the early (or late) stages of the disease leads to the failure to detect a correlation that may exist in the long-term progression of the disease. This limitation is not unique to PD and occurs in the neuroimaging research involving most neurodegenerative disorders.

In a limited number of studies, increased susceptibility was found in widespread cortical regions and correlated with cognitive impairment. Importantly, such involvement points to a cortical pathology in PD, however, no QSM study has examined dementia with Lewy bodies (DLB), a disorder characterized by a similar α-synuclein-related pathology. Future studies focussing on DLB are needed to examine the pattern of cortical iron alterations in synucleinopathies.

QSM studies have also examined susceptibility changes in other parkinsonian syndromes, such as PSP and MSA. PSP and MSA are progressive neurodegenerative diseases with parkinsonian features (such as tremor, rigidity, and abnormal movement). PSP is considered to be a tauopathy characterized by vertical gaze palsy, postural instability, rigidity, and cognitive impairment (Armstrong, 2018). According to two meta-analyses of morphometric studies in PSP, among the subcortical structures, the putamen, CN, thalamus, midbrain, and anterior cerebellum show significant atrophy in PSP (Shao et al., 2014; Pan et al., 2017). This involvement is reflected in the increased susceptibility shown by QSM in the SN, RN, and putamen. In the globus pallidus, however, although QSM shows higher susceptibility, such involvement has not been reported in morphometric studies.

MSA is an α-synucleinopathy (similar to DLB and PD) typically involving the striatum and SN, most commonly presents with Parkinsonian features and dysfunction of the autonomic system (Cykowski et al., 2015). Structural MRI studies have reported greatest gray matter reductions in the putamen and claustrum followed by the thalamus and cerebellum (Yu et al., 2015). In a similar fashion, QSM showed higher susceptibility in the SN and putamen, as well as the RN.

Clinical diagnosis and differentiation of the parkinsonian disorders is often challenging. In the limited number of studies reviewed, the pattern revealed by QSM in parkinsonian diseases showed high accuracy in their differentiation. This may suggest a clinical diagnostic utility for QSM in combination with other diagnostic tools. However, this remains to be more explored.

#### ALS

ALS is a progressive neurodegenerative disease characterized by the degeneration of upper and lower motor neurons in the motor cortex, brainstem and spinal cord. Copper-zinc superoxide dismutase (SOD1) mutation is one of the implicated genetic risk factors in ALS (Morgan and Orrell, 2016; Oskarsson et al., 2018). In animal models, it has been shown that SOD1 mutation results in increased expression of ferritin, transferrin receptor 1, and divalent metal transporter-1, leading to significant increase of iron in neural cells (Jeong et al., 2009). Additionally, even in the absence of SOD1 mutation, increased oxidative stress has been detected in patients with ALS, and suggested to play an important role in ALS pathogenesis (D'Amico et al., 2013). Further, in human studies, dysregulation in iron homeostasis has been reported in patients with ALS, including increased serum ferritin and decreased transferrin (Lovejoy and Guillemin, 2014).

ALS is commonly associated with MRI evidence for atrophy and reduced thickness in the motor cortex (precentral gyrus) (Cosottini et al., 2012; Chiò et al., 2014), and most QSM studies have focused on this region. Increased susceptibility was reported in these studies, which is in line with reports of increased oxidative stress in motor cortex and its correlation with disease severity (Ikawa et al., 2015). Further, T_2_, ${{\text{T}}_{2}}^{*}$, and SWI studies also support the finding of increased iron in the motor cortex of patients with ALS (Yu et al., 2014; Sheelakumari et al., 2016). An explanation for this iron accumulation can be the regional migration of iron-containing microglia and macrophages observed in post-mortem analyses (Kwan et al., 2012; Adachi et al., 2015).

The few studies investigating subcortical structures in ALS have reported inconsistent volume reductions in the basal ganglia and hippocampus (Grolez et al., 2016). One QSM study has examined these brain regions in ALS, showing increased susceptibility in the putamen, GP, RN and SN, and reduced susceptibility in the corticospinal tract (CST). Increased susceptibility in the basal ganglia is consistent with pathological findings in the nigrostriatal pathways in ALS (Takahashi et al., 1993), and transactive response (TAR) DNA binding protein 43 (TDP-43) pathology in the RN and striatum (Brettschneider et al., 2013). The CST is another key region involved in ALS (Toosy et al., 2003; Rajagopalan and Pioro, 2017), with its degeneration characterized by T_2_ hyperintensities (Goodin et al., 1988; da Rocha et al., 2004). Decreased susceptibility in the CST correlated with reduced fractional anisotropy and increased mean and radial diffusivity in DTI. These DTI changes usually indicate myelin degeneration and abnormalities. Thus, the QSM finding is surprising given that demyelination usually results in increased, rather than decreased susceptibility. Decrease in susceptibility may be due to other tissue microstructural changes, although this remains speculative (Takahashi et al., 1993; Brettschneider et al., 2013; Grolez et al., 2016; Acosta-Cabronero et al., 2018). Further, it is important to note that QSM measurement of susceptibility is dependent on the orientation of the neural fiber tracts and subject's head in relation to the scanner's magnetic field (Lee et al., 2017), which can pose a large confounding effect on QSM assessment of the white matter (Lancione et al., 2017).

#### Wilson's Disease

WD is an autosomal recessive condition, resulting from a mutation in the *ATP7B* gene, characterized by excessive deposition of copper within tissues. Most commonly affected organs are the brain and liver leading to neurodegeneration and hepatic damage, respectively. The most-affected brain regions are the basal ganglia, thalamus, cerebellum, and upper brainstem, leading to movement, cognitive and psychiatric impairments (Członkowska et al., 2018). QSM studies showed an increased susceptibility in subcortical structures including the putamen, GP, thalamus, SN and pons, reflecting their recognized pattern of involvement in both adult and pediatric patients with WD with neurologic involvement. Of note, the abnormal changes of susceptibility in the putamen, thalamus, and pons were also detectable in asymptomatic children with the WD genotype. This finding suggests that QSM can be a useful tool in exploring the complex pre-clinical pathophysiologic changes in WD.

It is not clear whether the increased susceptibility in the basal ganglia in WD is attributable to copper, iron or both. Although increased, the concentration of copper in the putamen, GP, and DN in patients with neurologic-WD is less than iron (Litwin et al., 2013; Dusek et al., 2017). There is an increased brain iron content in WD which results from dysfunction of the multicopper-dependent ferroxidase activity of ceruloplasmin. Ceruloplasmin is a multicopper containing protein that has a vital role in the export of iron from glial cells. In WD, the deficiency of *ATP7B* gene product leads to failure of incorporation of copper in ceruloplasmin (Członkowska et al., 2018). In the absence of functional ceruloplasmin, iron accumulates inside cells. In a study examining the correlation of iron and copper concentrations with ${{\text{R}}_{2}}^{*}$ mapping of susceptibility in post-mortem brain tissue from patients with WD, there was a strong correlation of ${{\text{R}}_{2}}^{*}$ measures with iron but not with copper concentrations (Dusek et al., 2017). Further, it is suggested that increased brain copper content in WD is likely in its diamagnetic Cu(I) state (Wender et al., 1974). Therefore, it is likely that increased magnetic susceptibility in WD results predominantly from secondary brain iron accumulation.

#### Huntington's Disease

HD is a genetic neurodegenerative syndrome, characterized by chorea, movement disorder and cognitive decline, and is caused by expansion of CAG trinucleotide repeats in exon 1 of huntingtin gene on chromosome 4. Evidence shows that abnormal huntingtin protein impairs iron homeostasis in the brain and is suggested to upregulate the expression of iron regulatory protein 1, transferrin, and transferrin receptor, which can result in increased iron accumulation (Niu et al., 2018). In mouse models of HD, increased production of mitochondrial iron uptake transporter (mitoferrin) and reduced frataxin lead to mitochondrial iron accumulation (Agrawal et al., 2018). Frataxin is a mitochondrial protein involved in the synthesis of iron-sulfur proteins necessary for oxidative phosphorylation in the mitochondria.

The brain structural hallmark of HD is degeneration of the CN and putamen and to a lesser extent, the GP, SN, STN, and locus coeruleus (Bhidayasiri and Truong, 2004). Higher iron content was reported in the basal ganglia by all of the studies in both patients with symptomatic HD and pre-symptomatic carriers of HD mutation, which correlated with atrophy in these structures, as well as age and genetic burden of HD. These findings are consistent with the recognized involvement of the basal ganglia in HD. Further, the increased iron content in asymptomatic individuals with the HD genotype is supportive of the findings from molecular studies indicating the association of abnormal huntingtin protein and dysregulation of iron homeostasis. Lower susceptibility was found in one study in the SN and hippocampus in patients with HD. The reason for this finding is not clearly understood but it may be associated with redistribution of iron within the brain (Van Bergen et al., 2016a).

In the cortical gray matter, structural studies have shown atrophy in the prefrontal and insular cortices (Lambrecq et al., 2013). Additionally, widespread deposition of huntingtin aggregates has been detected in the cortical regions including the insula, cingulate, and frontal cortices in clinical and preclinical HD (Gutekunst et al., 1999). These findings suggest a pathologic involvement of the cortex in HD in addition to the basal ganglia. To date, no QSM evaluation has been made in the cortical gray matter in HD. Such research would provide important information on the nature of cortical pathology in HD and verify whether a similar pattern of iron changes occurs in the cortex.

### Our Current Understanding, QSM Limitations and Future Directions

Measurements of magnetic susceptibility by QSM provide valuable information about the changes in tissue composition in the target regions. The existing literature suggests that QSM shows promise in the investigation of the pathophysiology of neurodegeneration by revealing changes in the gray matter, where considering the relative concentrations of paramagnetic and diamagnetic substances, increased susceptibility is most likely derived from increased levels of iron. In the white matter, however, due to the abundance of diamagnetic myelin, an increase in magnetic susceptibility may result from both demyelination and/or iron deposition (Langkammer et al., 2012; Sun et al., 2015; Hametner et al., 2018; Lee et al., 2018; Lewis et al., 2018; Wang et al., 2020). Further, susceptibility estimates in the white matter are often confounded by its geometrical properties, as the axonal orientation distribution with respect to the direction of the applied magnetic field has been shown to influence the inference of the true magnetic susceptibility distribution of the white matter tracts from the MRI phase images. Accounting for orientational dependence of white matter magnetic susceptibility estimates necessitates multiple orientation acquisitions or concurrent diffusion-weighted images, which can lead to longer scan times, subject discomfort and limited clinical feasibility (Liu, 2010; Lancione et al., 2017; Kaden et al., 2020).

Generally, studies using QSM showed increased susceptibility, suggestive of higher iron content, in the brain regions that are associated with the pathophysiology of each neurodegenerative disease, such as the SN in PD, the basal ganglia in HD, the amygdala and CN in AD, motor cortex in ALS and cerebellar DN in FRDA (Table 3). However, this pattern has not been consistently detected throughout all disorders, such as in AD where QSM did not reveal any changes in the hippocampus, which is recognized as one of the regions most involved by the pathologic processes in AD (Serrano-Pozo et al., 2011). One shortcoming of the reviewed papers on rare genetic neurodegenerative disorders is that most studies have restricted their scope of QSM evaluations to the iron rich basal ganglia and midbrain structures where susceptibility changes appear to be a common finding across some of these disorders (Table 3). A whole brain approach or investigation of a larger number of ROIs within all regions of the brain will provide a more exhaustive picture. It should also be noted that a number of relatively common neurodegenerative diseases, such as frontotemporal dementia, DLB, and prion diseases have not been investigated by QSM.

#### Methodological Aspects of QSM

QSM is an advanced post-processing technique for voxel-wise quantitative estimation of susceptibility, however, there are inherent limitations to the technique that restrict the robustness and reproducibility of susceptibility measurements by QSM. First, QSM estimates are sensitive to scanner field strength, acquisition parameters (such as echo time and voxel size), receiver noise, subject orientation and choice of QSM processing methods (Haacke et al., 2015). A description and critical comparison of these methods can be found in Deistung et al. (2017).

These dependencies lead to variations across studies in terms of the accuracy of measured susceptibilities. Another limitation of QSM is the surface artifacts (arising from strong susceptibility gradient at tissue interfaces, such as at the brain surfaces or around the blood vessels) and streaking artifacts (due to noise and mathematical limitations in solving the point-dipole problem) (Deistung et al., 2017; Taege et al., 2019; Jung et al., 2020). The third factor that contributes to the heterogeneity of susceptibility measurements across sites and studies is the inconsistent reference regions considered for relative measurement of magnetic susceptibility. In this review, we observed a diversity of reference regions used in studies including various white matter areas (frontal, occipital, etc.), CSF, or mean susceptibility value of the whole brain. Reporting the absolute susceptibility value without referencing has also been preferred in some studies. These discrepancies among referencing methods have made it difficult or impossible to directly compare the susceptibility values across different studies and disorders. Therefore, it is not currently feasible to perform a meta-analysis. QSM processing methods are under constant improvements, enhancing their robustness and reproducibility. Future studies using more robust and standardized QSM methods may enable more homogeneous and comparable measurements. Additionally, using a consensual referencing region in each neurodegenerative disease in future studies can be a step towards the comparison of QSM findings across studies. Finally, the possible effect of atrophy in measurements of mean susceptibility should not be overlooked. It has been suggested that atrophy of a ROI may result in higher iron concentration and therefore mean susceptibility in the absence of a change in absolute iron content (Taege et al., 2019). To account for and further explore this possibility, studies should consider the effect of atrophy as a confounder in magnetic susceptibility comparisons in their statistical analyses.

#### Future Directions

Although the small number of studies and the limitations of QSM make it difficult to draw definitive conclusions, the existing evidence suggests that QSM can greatly contribute to a better understanding of the underlying pathological changes in neurodegeneration. In most neurodegenerative diseases reviewed in this article, future studies with larger datasets, more consistent QSM referencing, and longitudinal designs are required. Multimodal studies using QSM and other neuroimaging techniques, such as PET can provide valuable information on the correlation of iron and pathologic protein aggregates. In the white matter, investigation of QSM together with diffusion weighted imaging allows more accurate examination of the alterations in myelin and iron. Finally, the high accuracy of QSM in differentiating certain neurodegenerative diseases, such as parkinsonian disorders, warrants further investigations aiming to evaluate its utility in clinical diagnosis of neurodegenerative diseases as an add-on imaging tool to the existing methods.

## Conclusion

Our review indicates that QSM provides evidence of altered iron distribution in the brain in neurodegenerative diseases. Currently, the number of QSM studies in most neurodegenerative diseases is limited, especially in the rare disorders. In PD that has been more widely studied by QSM, higher iron content in the SN as the most involved brain region has been revealed. The small number of studies in AD, ALS, and HD makes a confident conclusion out of reach. Nevertheless, this limited evidence is suggestive of disease-specific patterns of microenvironmental changes that are worthy of further investigation. This *in-vivo* evidence is in line with the reports from cellular and molecular studies (suggesting a pathologic role for iron in neurodegeneration) and post-mortem studies (indicating increased iron content in the brain in these disorders) (Zecca et al., 2004). Further, the increased burden of iron correlates with cognitive deficits in AD, disease stage in PD, and genetic burden and age in HD. Such associations may point out ongoing relationships between iron accumulation and neurodegeneration, either causal, intermediate, or independent.

Based on the findings of this review, QSM provides a unique opportunity for investigation of neurodegenerative diseases that can improve our understanding of the pathologic processes involved.

## Data Availability Statement

The original contributions presented in the study are included in the article/Supplementary Material, further inquiries can be directed to the corresponding author/s.

## Author Contributions

PR developed the search strategies, ran the database search. PR and SML, have each independently performed the screening of studies for inclusion, data extraction, and quality assessment. CP adjudicated the conflicts between two reviewers, PR and SML, in screening, data extraction, and quality assessment. Development of the protocol was done by PR, and all authors have collaborated to improve, and approved the final protocol. Data synthesis was performed by PR and SML. CP, DV, AIB, and BAM provided the intellectual input to data synthesis and interpretation. VLC, WTS, PD, AIB, BAM, DV, CP, TEVR, CMO, DJRL, and SML provided the intellectual input into the manuscript draft prepared by PR. All authors have read, provided feedback, and approved the final manuscript.

## Conflict of Interest

The authors declare that the research was conducted in the absence of any commercial or financial relationships that could be construed as a potential conflict of interest.

## Supplementary Material

Supplementary Material 1 Protocol for systematic review.

Supplementary Material 2 Tables S1A–H. Summary of the characteristics, findings and risk of bias assessment of QSM studies in neurodegenerative diseases.

Supplementary Material 3 Results and discussion sections on less studied neurodegenerative disorders.

Supplementary Material 4 Table S2. Details of MRI acquisition and QSM processing methods used by reviewed studies.

## Abbreviations

Aβ: Amyloid-β
AD: Alzheimer's disease
ALS: Amyotrophic lateral sclerosis
ALSFRS: Amyotrophic lateral sclerosis Functional Rating Scale
aMCI: amnestic mild cognitive impairment
AR-PD: akinetic-rigid Parkinson's disease
AUC: area under curve
CN: caudate nucleus
CNS: central nervous system
CSF: cerebrospinal fluid
CST: corticospinal tract
DLB: dementia with Lewy bodies
DM1: myotonic dystrophy 1
DM2: myotonic dystrophy 2
DN: dentate nucleus
DTI: diffusion tensor imaging
FD: Fabry disease
FRDA: Friedreich's ataxia
GP: globus pallidus
GRE: gradient (recalled) echo
GRE: gradient echo
H&Y: Hoehn and Yahr
HD: Huntington's disease
iLSQR: iterative LSQR
LSQR: sparse linear equation and least-squares
MCI: mild cognitive impairment
MEDI: morphology enabled dipole inversion
MMSE: mini-mental status examination
MoCA: Montreal cognitive assessment
MPAN: mitochondrial membrane protein-associated neurodegeneration
MRI: magnetic resonance imaging
MSA: multiple system atrophy
NBIA: neurodegeneration with brain iron accumulation syndromes
nMEDI: non-linear MEDI
PD: Parkinson's disease
PDF: projection onto dipole fields
PET: positron emission tomography
PKAN: pantothenate-kinase-associated neurodegeneration
PLS: progressive primary lateral sclerosis
PSP: progressive supranuclear palsy
QSM: quantitative susceptibility mapping
RE-SHARP: regularization-enabled SHARP
RN: red nucleus
ROC: receiver operating characteristic curve
SCA: spinocerebellar ataxia
SHARP: sophisticated harmonic artifact reduction for phase
SN: substantia nigra
SNc: substantia nigra pars compacta
SNr: substantia nigra pars reticulata
SOD1: superoxide dismutase 1
STN: sub-thalamic nucleus
SWI: susceptibility weighted imaging
TD-PD: tremor-dominant Parkinson's disease
UPDRS: unified Parkinson's disease rating scale
V-SHARP: variable-radius SHARP
WD: Wilson's disease.
