# Supplementary material for: Systematic Review: Quantitative Susceptibility Mapping (QSM) of Brain Iron Profile in Neurodegenerative Diseases
Source: Front Neurosci. 2021 Feb 18;15:618435. doi: 10.3389/fnins.2021.618435 (PMC7930077; doi:10.3389/fnins.2021.618435)
Supplement: Supplementary Material 2 — Tables S1A–H. Summary of the characteristics, findings and risk of bias assessment of QSM studies in neurodegenerative diseases. [file Table_2.DOCX]

**Supplementary material 2. Table S1**

| **Table S1a. Summary of QSM studies in AD** | | | | | | | | |
| --- | --- | --- | --- | --- | --- | --- | --- | --- |
| **Study** | **Field strength** | **Modality** | **ROI** | **Sample size** | **Disease stage** | **Mean age ± SD (y)** | **Main findings** | **Risk of bias** |
| Acosta-Cabronero et al., (2013) | 3T | QSM | Voxel-based,  ROI-based: CN, PUT, GP, TH, HP, AMY | Early-stage probable AD: 8 HC: 8  Young HC: 3 | MMSE: 22 ± 4 | AD: 72 ± 6 HC: 70 ± 5 | - Higher χ in bilateral PUT, left AMY and right CN - No association between HP atrophy and χ in the PUT - Scattered clusters of higher χ in occipitoparietal and temporoparietal grey and white matter regions | medium |
| Van Bergen et al., (2016) | 7T | QSM, amyloid PET, fMRI | AMY, NA, HP, Entorhinal cortex, TH, CN, PUT, GP, Frontal, Temporal, parietal and occipital cortex | aMCI: 15 HC: 22 | MMSE:  ﻿28.61 ± 1.65  MoCA:  ﻿24.44 ± 2.17 | HC: 71.91 ± 5.25  aMCI: 75.27 ± 7.63 | - No difference of χ between MCI and HC in any of the ROIs - In the MCI group, APOE-e4 carriers showed significantly higher χ in CN and frontal, parietal, temporal and occipital cortices. - In the MCI group, in the cortical regions showing altered coupling with medial prefrontal cortex, χ significantly correlated with Aβ deposition. | Low |
| Moon et al., (2016) | 3T | QSM | PUT, GP, CN, PUL | VaD: 12 probable AD: 27 HC: 18 | MMSE AD:  14.70 ± 5.81  MMSE VaD:  15.17 ± 7.04 | VaD: 79.92 ± 6.80 AD: 78.63 ± 8.11 HC: 46.89 ± 14.69 | - In both AD and VaD, PUT and CN showed higher χ than controls. - χ did not correlate with age and severity of cognitive deficits (MMSE and CDRSOB) | Low |
| Hwang et al., (2016) | 3T | QSM texture analysis | Whole brain texture analysis,  ROIs:  Basal Ganglia, HP, PUT | HC: 18 aMCI: 18 AD: 18 | MMSE AD: 17.56 ± 3.5  MMSE aMCI:  ﻿27.61 ± 2.17 | HC: 65.2 ± 6.41  aMCI: 66.9 ± 5.51 AD: 69. 9 ± 9.81 | - Higher mean χ of whole white matter in AD vs. controls. - No difference of mean χ in the basal ganglia, HP, and PUT among groups - Several first- and second-order texture parameters were able to differentiate aMCI subjects from HC group. | Medium |
| Ayton et al., (2017) | 3T | QSM, amyloid PET | HP, Frontal lobe, Temporal lobe, Parietal lobe,  Occipital lobe, Cingulate, CN | HC: 64 MCI: 17 AD:19 | Not reported | Aβ+: 76.4 ± 1.0 Aβ-: 74.0 ± 0.9 | - Colocalization of higher χ and Aβ accumulation in the frontal lobe, temporal lobe, occipital lobe, but not in the parietal lobe, HP, cingulate gyrus or CN - No group difference in QSM in any of the ROIs when Aβ SUVR was also included in the modelling. - In Aβ+ subjects: Higher χ in the HP predicted greater impairment in episodic memory, executive function and attention. - Higher χ in the temporal lobe and frontal lobe were associated with declining of performance in a composite of language tests. | Low |
| Kim et al., (2017) | 3T | QSM, grey matter volume | Voxel-based analysis.  ROIs: AMY, GP,  HP, precuneus,  PUL, PUT, TH.  Amyloid-deposition-based ROIs: neocortex, allocortex, entorhinal cortex, anterior and posterior cingulate cortex | HC: 19 aMCI: 19 AD: 19 | MMSE aMCI:  ﻿27.63 ± 2.11  MMSE AD:  ﻿17.37 ± 3.42 | HC: 65.37 ± 6.29 aMCI: 65.95 ± 6.75 AD: 69.79 ± 10.27 | - Voxel-based: Increased χ in ﻿lt. and rt. precentral gyri, lt. and rt. postcentral gyrus, rt. superior temporal gyrus, rt. Amygdala, lt. posterior cingulate, lt. HP, rt. transverse temporal, rt. supramarginal gyrus, rt. parahippocampal gyrus, lt. inferior parietal lobule, rt. middle frontal gyrus, rt. cingulate gyrus, rt. and t. insula, lt. CN tail, rt. CN body, and rt. culmen in anterior lobe in AD vs. HC - ROI-based: Higher χ in the HP, AMY, precuneus, TH, neocortex, allocortex, entorhinal cortex, and anterior and posterior cingulate cortex in AD vs. HC - Increased QSM in the AMY and neocortex in AD compared to aMCI - Increased QSM in the precuneus, allocortex, and anterior and posterior cingulate cortex in aMCI vs. HC | Low |
| L. Du et al., (2018) | 3T | QSM | ﻿CN, PUT, GP, TH, RN, SN, DN | HC: 30  Mild-Moderate AD: 30 | MMSE: ﻿20.4 ± 2.4  MoCA: ﻿20.4 ± 3.2 | HC: ﻿66.2 ± 7.8  AD: ﻿68.3 ± 6.6 | - Higher χ in bilateral CN and PUT - Correlation of MMSE and MoCA with χ in the left CN - Lower χ in bilateral RN in AD | Low |
| Tiepolt et al., (2018) | 7T | QSM,  Amyloid PET | GP, PUT, Cortical regions | HC: 10  AD: 10 | MMSE: ﻿23.6 ± 7.3 | HC: 67.1  AD: 74.1 | - Higher χ in bilateral GP in AD - χ of the GP correlated with cognitive function assessed by MMSE - No correlation of QSM and amyloid-PET in the GP | Low |
| Van Bergen et al., (2018) | 3T | QSM, Amyloid-PET | Whole brain | 116 elderly individuals with normal cognition | MMSE: ﻿28.99 ± 1.10 | ﻿74.81 ± 7.52 | - Significant correlation between amyloid-PET and increased χ in QSM was found in 36 voxel clusters in the cortex and subcortical regions, strongest correlation being in GP, CN and PUT. | Low |
| Meineke et al., (2018) | 3T | QSM | GP, CN, PUT, TH, HP | Mild to moderate AD: 6  MCI: 8  HC: 10 | MMSE AD:  ﻿19.2 ± 3.2  MMSE MCI:  ﻿25.6 ± 2.1 | AD: ﻿58 ± 6  MCI: ﻿63 ± 6  HC: ﻿59 ± 7 | - Higher mean χ in the CN and PUT in patients with AD compared to controls - No significant difference was found between MCI and control groups | Medium |
| Kan et al., (2020) | 3T | QSM | Voxel-based comparison | HC: 19  AD: 38 | Not reported | HC: 71 ± 5  AD: 80 ± 6 | - Higher χ in the HP, AMY, and CN in AD | Medium |
| Kagerer et al., (2020) | 3T | QSM, amyloid-PET, BOLD | Whole brain association of DMN and χ | APOE4 + HC: 18  AOE4 – HC: 51 | MMSE APOE4+:  ﻿29.12 ± 1.58  MMSE APOE4-:  ﻿29.4 ± 0.89 | APOE4 + HC: ﻿66.28 (5.29)  APOE4 – HC: ﻿66.04 (7.87) | - No difference between whole cortical χ among APOE4 positive and negative groups. - Positive correlation of iron content and DMN activity that correlates with APOE4 status most prominent in precuneus, posterior cingulate and lateral parietal cortex. | Low |
| Tuzzi et al., (2020) | 9.4 T | QSM | Frontal cortex | AD: 2  HC: 2 | Not reported | Not reported | - χ values up to 40 ppb were observed in the cortex of patients with AD while the maximum cortical χ in HC group was 25 ppb. - No statistical analysis among groups | High (statistical limitation) |
| **Table S1b. Summary of QSM studies in PD** | | | | | | | | |
| **Study** | **Field Strength** | **Modality** | **ROI** | **Sample size** | **Mean age ± SD (y)** | **Disease duration** | **Main findings** | **Quality assessment** |
| Lotfipour et al., (2012) | 7T | Susceptibility mapping | SN, SNc | PD: 9  HC: 11 | PD: 66.6  HC: 59.2 | Not provided | - Increased χ in the SNc in PD group but this significance was lost after controlling for age. - Significant susceptibility gradient from higher in caudal to lower in cranial SN in both PD and HC subjects. | High (inconsistent MRI acquisition due to scanner upgrade) |
| Ide et al., (2014) | 3T | QSM, R2* | GP, lateral GP (GPl), medial GP (GPm) | PD: 19  HC: 41 | PD: 72.6 ± 7.65  HC: 69.4 ± 9.10 | Not provided | - No significant difference in χ of GPl, GPm, or whole GP among patient and controls. | Low |
| Barbosa et al., (2015) | 3T | QSM | SN, SNc, RN, GP, PUT, CN, TH, WM, GM | PD: 20  HC: 30 | PD: 66 ± 8  HC: 64 ± 7 | PD: 8.1 y | - Higher χ in the SN and SNc in PD - No correlation between χ and disease duration | Low |
| He et al., (2015) | 3T | QSM, T2* | Head of CN, PUT, GP, SN, RN | PD: 44  HC: 35 | PD: 58 ± 8.76 HC: 60.49 ± 6.48 | PD: 2.82 ± 1.64 y | - Increased χ in the SN bilaterally, and RN contralateral to the more affected side in PD - A trend towards decreased χ in PUT in PD | Low |
| Murakami et al., (2015) | 3T | QSM, R2* | TH, SN, PUT, CN, GP, RN | PD: 21  HC: 21 | PD: 72.0 ± 7.5 HC: 69.7 ± 8.6 | PD: 32.7 ± 27.1 m | - In patients with PD, the SN showed significantly higher χ compared to controls. - QSM showed high accuracy in differentiating PD from control groups (sensitivity: 90%, specificity: 86%). | Medium |
| Azuma et al., (2016) | 3T | QSM | SN (subfields), GP, RN, PUT, CN | PD: 24  HC: 24 | PD: 63.3 ± 11.0 HC: 64.1 ± 10.0 | PD: 77.1 ± 43.7 m | - χ in the middle and posterior SN, and whole SN was significantly higher in patients with PD than in the healthy controls. - A trend toward lower χ in GP and PUT in PD | Low |
| Peckham et al., (2016) | 3T | QSM | SN, FN, GM, PUT, TH, RN, DN | PD: 18  HC: 16 | PD: 69.1 ± 11.2 HC: 64.4 ± 6.1 | PD: 1.1 ± 0.3 m | - Significantly increased χ in the SN in PD - Other ROIs showed no significant difference among PD and HC groups. - A trend of higher maximum and larger standard deviation of susceptibility in the fasciculata nigrale was observed in the PD group. - In all subjects, a pattern of increasing χ along the fasciculata nigrale with higher χ at its caudal region was noted. | Low |
| Langkammer et al., (2016) | 3T | QSM, R2* | SN, SNc, SNr, CN, GP, PUT, TH | PD: 66  HC: 58 | PD: 64.7 ± 8.8  HC: 65.0 ± 9.3 | PD: 3.4 (0.25 -24.9) y | - Higher χ in the SN, RN, TH, and GP in PD - χ of all ROIs except RN was correlated with one or more clinical markers of motor and non-motor disease severity (H&Y stage, total UPDRS, UPDRS-I and II). - No correlation of χ in any of the regions with disease duration | Low |
| Du et al., (2016) | 3T | QSM, R2* | Voxel-wise analysis of midbrain,  ROI analysis: SNc | PD: 47  HC: 47 | PD: 65.8 ±10.1  HC: 62.2 ± 8.8 | PD: 5.5 ± 4.8 y | - Significantly higher χ in SN and SNc in PD - QSM in the SNc correlated with LEDD, disease duration and UPDRS II, but not UPDRS-I and III. | Low |
| He et al., (2017) | 3T | QSM | DN (cerebellum) | PD: 43  (AR: 24; TD: 19)  HC: 48 | PD: 63.7 ± 6.9  HC: 61.7 ± 6.5 | TD: 4.2 ± 3.9 y AR: 7.0 ± 4.5 y | - Higher χ in the DN in TD-PD compared to both HC and AR-PD. - No variation in χ of the DN among HC and AR-PD groups. - Higher χ in ipsilateral DN correlated with higher tremor score. | Low |
| Ito et al., (2017) | 3T | QSM, DKI | Anterior and posterior PUT, GP | PD: 26  MSA-P: 6  MSA-C: 7  PSP: 14  HC: 20 | PD: 64  MSA-P: 67.5  MSA-C: 72  PSP: 68.5  HC: 68.5 | PD: 1.5 y  MSA-P: 1.9 y  MSA-C: 1.5 y  PSP: 1.5 y | - χ was significantly higher in the posterior PUT in MSA-P, and in the anterior PUT and GP in patients with PSP compared to PD. - There was no significant variation of χ in any of the regions between HC, PD, and MSA-C groups. - ROC analysis revealed high accuracy of χ in the posterior PUT in differentiation of PD from MSA-P groups. | Medium |
| Sjöström et al., (2017) | Mixed data from 3T and multiple 1.5T scanners | QSM | PUT, GP, SN, RN | PD: 62  PSP: 15  MSA: 11  HC: 14 | PD: 65.2 ± 10.5  PSP: 69.1 ± 6.0  MSA: ﻿68.9 ± 13.1  HC: 63.5 ± 5.3 | PD: 4.7 ± 4.4 y  PSP: 2.8 ± 1.0 y  MSA: 3.6 ± 2.7 y | - Patients with PSP had increased χ in the PUT and GP compared to all other groups, and in the SN compared to the HC group. - Patients with MSA showed higher χ in the PUT and RN compared to both PD and HC groups, and in the SN compared to HC group. - χ of the SN was higher in PD group compared to the healthy controls. | High (various MRI acquisition protocols) |
| Xuan et al., (2017) | 3T | QSM | GP, PUT, head of CN, SNc, SNr, RN | EOPD: 35 younger HC: 24  M-LOPD: 33  Older HC: 22 | EOPD: 50.0 ± 5.3  Young HC: 51.8 ± 7.5  M-LOPD: 61.9 ± 6.0  Elderly HC: 63.1 ± 7.3 | EOPD: 4.4 ± 3.4 y LOPD: 3.7 ± 2.8 y | - Both patient groups had increased χ in the SNc and SNr, but the M-LOPD group also showed increased χ in the PUT. - The increased χ in the SNc, SNr and PUT positively correlated with the disease severity (H&Y stages, UPDRS II scores and UPDRS III scores) in the M-LOPD patients, which was not observed in the EOPD patients | Low |
| Guan et al., (2017b) | 3T | QSM, R2* | GP, PUT, head of CN, SNc, SNr, RN, TH, DN | AR-PD: 27  TD-PD: 27  HC: 40 | AR-PD: 55.4 ± 9.9  TD-PD: 55.3 ± 9.0  HC: 56.6 ± 9.9 | AR-PD: 4.0 ± 2.8 y TD-PD: 5.4 ± 4.7 y | - Increased χ in the SNc in both PD groups compared to HC but no difference among PD groups. - Higher χ in the RN and DN in TD-PD than HC and AR-PD. - H&Y stage and motor scores of UPDRS correlated with χ in the SNc. - Tremor severity scores correlated with RN and χ in the SNc. - Akinetic/rigid scores were associated with χ in the CN. | Low |
| Guan et al., (2017a) | 3T | QSM, R2* | GP, PUT, head of CN, SNc, SNr, RN, DN, TH | ES-PD: 15  LS-PD: 45 HC: 40 | ES-PD: 55.8 ± 8.3  LS-PD: 61.1 ± 6.7  HC: 56.6 ± 9.9 | ES-PD: 4.6 ± 3.7 y LS-PD: 6.7 ± 4.7 y | - Comparing ES-PD with HC, the SNc showed higher χ. - In later stages, increased χ was observed in the SNr, GP and RN in addition to SNc. - χ in the SNc was significantly higher in LS-PD compared to both HCs and ES-PD. - χ of GP and SNc correlated with disease severity. | Low |
| Zhao et al., (2017) | 3T | QSM, R2* | SN, RN, PUT, CN, GP | PD: 29  HC: 25 | PD: 67.9 ± 6.7 HC: 64.7 ± 8.3 | Not provided | - Significantly higher χ in the SN in PD patients - No significant variation between χ of other ROIs among two groups - No correlation between UPDRS -III and QSM in any of the ROIs | Medium |
| Acosta-Cabronero et al., (2017) | 3T | QSM | Whole brain voxel-wise analysis;  ROIs: CN, GP, PUT, HP, AMY, TH, RN, SN, DN, lateral occipital, middle temporal, posterior parietal, rostral middle prefrontal cortex, precentral and postcentral gyri | PD: 25  HC: 50 | PD: 63.6 ± 8.6  HC: 63.6 ± 8.5 | PD: 6 ± 4 y | - Increased bulk χ in rostral pontine areas and in a cortical pattern closely concordant with known Parkinson's disease distributions of α-synuclein pathology. - Reduced χ in the DN in PD. - Higher regional QSM in the SN, along with lateral occipital, posterior parietal and rostral middle prefrontal cortices in PD. Less pronounced increase in QS in middle temporal gyrus and HP. - No association between QSM and clinical features of PD (UPDRS-III and MMSE). - In healthy controls, χ increased with age in the PUT, middle temporal and prefrontal structures. | Low |
| Takahashi et al., (2018) | 3T | QSM and NM-MRI | SNc | PD: 39  HC: 25 | PD: 69.24 ± 6.04  HC: 67.14 ± 6.81 | PD: 5.24 ± 3.56 y | - SNc χ value was significantly higher in patients with PD compared to HCs. - Neuromelanin value was significantly lower in PD. | Medium |
| Kim et al., (2018) | 3T | QSM combined with histogram analysis | SN | ES-PD: 38  HC: 25 | ES-PD: 68.3 ± 9.2  HC: 65.0 ± 6.3 | ES-PD: 12 (4.5–22.5) m | - Higher mean χ of lower SN parts in early-stage idiopathic PD compared to HCs - χ of lower SN yielded area under ROC curve of 0.932 suggestive of high accuracy in differentiation of early stage PD from HC. | Low |
| Du et al., (2018) | 3T | Longitudinal QSM, R2* | SNc, SNr, RN | PD: 72  HC: 62 | PD: 66.3 ± 9.5  HC: 66.2 ± 10.2 | PD: 4.5 ± 4.5 y | - Higher χ in the SNr and SNc in PD at baseline. - χ of SNc showed significant increase in PD group in R2* maps but not in QSM. - χ of SNr decreased in QSM and R2* maps during the follow up in both PD and HC groups. - No difference in χ of the RN among groups at any time. - χ in the SNr correlated with UPDRS-III - No correlation between χ in any of the regions with UPDRS-I, UPDRS-II, MoCA, and PDQ-39. | Low |
| Shin et al., (2018) | 3T | QSM | RN, SNc, SNr, DN, GP, PUT, head of CN | ES-PD: 29  (High-NMS: 13,  Low-NMS: 16)  HC: 19 | High NMS PD: 69.8 ± 11.2  Low NMS PD: 71.4 ± 6.8  HC: 67.6 ± 8.0 | High-NMS PD: 67.9 ± 11.4 m Low-NMS PD: 70.4 ± 6.5 m | - No difference of χ was detected among PD and HC groups. - There was no significant correlation between χ in any of the ROIs and disease duration, motor and non-motor symptoms severity scales. | Low |
| Takahashi et al., (2018) | 3T | QSM and neuromelanin area volume | SNc | ES-PD: 18  HC: 18 | ES-PD: 71.2 ± 6.94  HC: 67.1 ± 4.75 | PD: 5.39 ± 1.95 y | - PD: higher χ and smaller NM area in SNc bilaterally. - Diagnostic specificity and sensitivity were 0.70/0.73 for the QSM and 0.81/0.78 for the neuromelanin area in the whole/dorsolateral SNc | Medium |
| An et al., (2018) | 3T | QSM | SN | PD: 44  HC: 31 | PD: 67.3 ± 9.8 HC: 66.9 ± 9.0 | PD: 4.1 ± 2.0 y | - Higher χ in the SN in PD which correlated with H&Y stage, UPDRS, MADRS, and HAMA score. - Significantly higher χ in the SN in both AR-PD and TD-PD compared to controls. - No difference in χ in the SN among TD and AR subgroups | Low |
| Li et al., (2018) | 3T | QSM | SN, CN, PUT, GP, AMY, HP, TH, RN, DN | PD with dementia (PDD): 10  PD without dementia: 31 HC: 27 | PDD: 72.6 ± 5.8 PD: 63.1 ± 8.3  HC: 62.0 ± 7.0 | PDD: 12.8 ± 8.1 y  PD: 7.6 ± 4.6 y | - PDD group had higher χ in lt. HP compared to PD group, and in bilateral HP compared to HC group. - PD group had higher χ in the rt. TH and rt. HP compared to HC. - χ in the rt. and lt. HP inversely correlated with MMSE scores. | Low |
| Mazzucchi et al., (2019) | 3T | QSM | SN, RN, STN, PUT, GP, CN | PD: 35  MSA: 12  PSP:13 | PD: ﻿61.0 ± 8.6  MSA: ﻿65.6 ± 8.1  PSP: ﻿70.3 ± 5.9 | PD: ﻿4.7 ± 6.3 y  MSA: ﻿3.1 ± 1.9 y  PSP: ﻿3.8 ± 1.6 y | - χ values were highest in the PUT in the MSA group, and in the RN, STN, and medial SN in the PSP group which differentiated each group from another and the PD group with high accuracy. - Compared to PD, χ was higher in the SN, PUT, STN, and RN in both PSP and MSA groups. - Higher χ was observed in the SN and RN in PSP compared to MSA. | Low |
| Miyata et al., (2019) | 3T | QSM | dorsal and ventral precentral gyrus, dorsal and ventral postcentral gyrus, superior frontal gyrus, middle frontal gyrus, and occipital gyrus | CBD: 12  PSP: 14  PD: 30  HC: 30 | CBD: ﻿72.4  PSP: ﻿75.8  PD: ﻿70.7  HC: ﻿56.3 | CBD: ﻿3.2 y  PSP: 2.5 y  PD: 4.1 y | - A three-layer appearance (consisted of hyperintensity in the superficial layer of the cortex and corticomedullary junction with a layer between them with lower χ) was observed in the cortex of 83 percent of CBD patients, 21 percent of PSP patients and none of PD patients. - A similar three-layer appearance was also noticed in the histo-pathologic analysis of a CBD post-mortem brain sample with ferritin staining. | Low |
| Guan et al., (2019a) | 3T | QSM, DTI | White matter voxel-wise tract-based analysis | PD: 65  HC: 46 | PD: 55.5 ± 9.5  HC: 57.8 ± 9.4 | PD: 4.7 ± 3.9 y | - Increased χ of white matter regions in the frontal, parietal, and temporal lobes, including bilateral external capsule, inferior longitudinal fasciculus, inferior fronto-occipital fasciculus, left superior longitudinal fasciculus, right anterior thalamic radiation, left cingulum, and the body of the corpus callosum in PD. - On the other hand, χ in bilateral deep white matter of the frontal lobes and right cerebellar hemisphere were decreased in PD - χ of the rt. ILF correlated with UPDRS total score and the rt. cingulum with disease duration. - ILF was the only region that showed changes in χ and DTI both. | Low |
| Li et al., (2019) | 3T | QSM (texture analysis), R2* | SN | PD: 28  HC: 28 | PD: 68.20 ± 6.1  HC: 64.75 ± 8.0 | not reported | - Texture analysis of QSM revealed significantly higher mean χ with more homogeneous distribution of χ within the SN in PD compared to controls. | Low |
| Azuma et al., (2019) | 3T | QSM | SN, RN, PUT, CN, GP | PD: 18  PSP: 8  HC: 18 | PD: 69.6 ± 6.2  PSP: 69.5 ± 7.7  HC: 69.1 ± 7.0 | PD: 74.6 ± 54.0 m  PSP: 40.5 ± 40.3 m | - χ of the GP and SN was higher in PSP compared to PD. - Compared to HC, χ was higher in the GP, SN, and RN in the PSP group. - χ of the SN was higher in PD compared to HC. - ROC curve analysis showed high accuracy (AUC: 0.903) of GP χ in differentiation of PD from PSP. | Low |
| Guan et al., (2019b) | 3T | QSM, resting state fMRI, DTI | SN QSM, and functional connectivity among SN, Striatum, GP, and TH | PD: 90  HC: 38 | PD: 59.38 ± 8.54 HC: 57.93 ± 8.04 | PD: 3.97 ± 4.09 y | - Increased χ in the inferior parts of the SN and SNc in PD, but no significant changes in χ in the middle and upper parts. - χ in inferior SN and SNc correlated with PDQ-39 symptom severity scale. - Nigral χ significantly correlated with functional connectivity measures. | Low |
| Shahmaei et al., (2019) | 3T | QSM | RN, CN, SN, GP, PUT, TH | PD: 30  HC: 15 | PD: 66.2 ± 8.5  HC: 64.9 ± 9.2 | Not provided | - Patients with PD showed higher χ in the RN, SN, GP, TH. - No significant correlation of χ with age. - Significant correlation of χ in the SN, RN and GP with disease H&Y stage. - High sensitivity (100%) and specificity (93%) for χ of the SN in differentiation of PD from HC. | Medium |
| Sethi et al., (2019) | PD: 3T HC: 1.5T | QSM | RN, SN | PD: 20  HC: 174 | PD: 67.0 ± 10  HC: 45.1 ± 14.2 | PD: 7.1 ± 5 y | - Higher mean χ in whole-region and threshold high-iron region in SN in PD. - Higher mean χ in threshold-high iron region but not the whole-region of RN. - (Threshold high-iron regions= voxels “﻿lying higher than the upper 95% prediction intervals in the susceptibility-age whole-region analysis”) | High (age-matching not performed, inconsistent MRI acquisition) |
| Uchida et al., (2019) | 3T | QSM | Voxel-based whole brain analysis;  ROIs-based: SN, GP, PUT, RN, CN, TH, HP, AMY, entorhinal cortex, orbitofrontal cortex, parahippocampal gyrus, and precuneus | PD-MCI: 24  PD: 22  HC: 20 | PD-MCI: 74.9 ± 5.5 PD: 70.8 ± 5.9 HC: 71.4 ± 5.2 | PD-MCI: 7.2 ± 4.4 y  PD: 7.3 ± 3.4 y | - PD-MCI group had higher χ in the CN and AMY as well as entorhinal, parahippocampal, and precuneus cortical regions compared to PD group. - Compared to HC group, χ was higher in the SN, GP, and PUT in patients with PD. - Patients with PD with MCI had increased χ in the orbitofrontal cortex compared to controls. - Significant negative correlation was found between MoCA score and χ in the CN and cuneus. - χ of the AMY, cuneus, and fusiform gyrus correlated with Open Essence olfaction test. - χ of the PUT positively correlated with UPDRS-III scores in patients with PD. | Low |
| Sun et al., (2019) | 3T | QSM | SN, GP, RN, head of the CN, and PUT | iRBD: 25  PD: 32  HC: 50 | iRBD: 62.5 ± 6.1  PD: 61.6 ± 6.5  HC: 62.0 ± 7.5 | iRBD: 4.2 ± 2.6 y  PD: 4.0 ± 3.4 y | - In patients with PD, χ was higher in the SN compared to both idiopathic REM sleep behaviour disorder (iRBD) and HC groups, and in the GP and lt. RN compared to HC. - iRBD showed higher χ in the SN compared to healthy controls. - χ of the SN differentiated iRBD from PD with high accuracy (90.4%). | Medium |
| Cheng et al., (2019) | 3T | QSM | Nigrosome-1 | PD: 87  HC: 77 | PD: 60.9 ± 8.1  HC: 63.4 ± 7.3 | Not provided | - Nigrosome-1 region was smaller in PD group and had higher χ. - Using the 105 radiomic features from QSM significantly improves the accuracy of PD diagnosis. | Low |
| Ghassaban et al., (2019) | 3T | QSM | PUT, GP, TH, PUL, SN, DN, head of CN | PD: 25  HC: 24 | PD: 61.8 ± 6.4  HC: 63.4 ± 8.0 | Not provided | - Significantly higher χ of the SN in PD group. | Medium |
| Wang et al., (2019) | 3T | QSM, DTI, R2* | HP, AMY, NA | PD: 35 MSA-P: 16 PSP: 17 HC: 37 | PD: 71.0 ± 7.4 MSA-P: 66.4 ± 8.2 PSP: 72.5 ± 9.7 HC: 70.4 ± 7.8 | PD: 3.4 ± 3.6 y MSA-P: 4.1 ± 3.3 y PSP: 3.3 ± 2.9 y | - No difference in QSM values in any of investigated regions among Parkinsonian diseases, nor between the disease states and controls. | Low |
| Bergsland et al., (2019) | 3T | Longitudinal QSM (two time points  3 years apart) | SN | PD: 18 HC:16 | PD: 60.1 ± 6.2 HC: 58.1 ± 8.7 | PD: 6.2 y | - Higher χ in the ventral posterior and anterior SN in PD. - Significant increase in χ after 3 years was found only in the ventral posterior SN in PD, but not the HC group. | Low |
| Hwang et al., (2019) | 3T | QSM, DWI | Voxel-wise: Cortical GM  ROI-based: NA, TH, PUT, AMY, GP, CN, HP, brainstem | PD-Hyposmia: 62 PD-Normosmia: 40 | PD-Hyposmia: 65.7 ± 7.85 PD-Normosmia: 65.7 ± 10.4 | PD-Hyposmia: 0.5 ± 1.5 y PD-Normosmia: 0.5 ± 2.4 y | - χ was increased in the lt. TH, while decreased the rt. TH in PD patients with hyposmia. - No variation of χ was found among the groups in the cortex. | Low |
| Chen et al., (2019) | 3T | QSM | SNc, SNr, FN, GPi, GPe, STN, RN, PUT, CN, TH, DN, cortex | PD: 33 HC: 26 | PD: 64.55 ± 11.2 HC: 62.62 ± 10.62 | PD: 2.77 ± 2.41 y | - PD group showed increased χ values in the FN, SNc, GPi, RN, PUT and CN compared to the HC group - The SNc, SNr and RN showed significantly increased χ values in patients with LS-PD compared to ES-PD. - Mean χ of the whole cortex did not show any difference among groups. | Low |
| Cheng et al., (2020) | 3T | QSM | SN, nigrosome-1 | PD: 57  HC: 80  Atypical parkinsonism: 14  Essential tremor: 9 | Parkinsonian disorders: 63.8 ± 8.6  HC: not provided | Not provided | - In patients with PD who had bilateral loss of nigrosome-1, χ of the SN was significantly higher than HC group -who had bilaterally visible nigrosome-1. - In patients with PD who had nigrosome-1 region visible bilaterally or unilaterally, there was no difference of χ in the SN from controls. - In atypical parkinsonism patients with both bilateral and unilateral loss of nigrosome-1, χ of the SN was higher compared to controls. - In the essential tremor group, Nigrosome-1 was visible bilaterally and showed no difference of χ compared to controls. | Medium |
| Ahmadi et al., (2020) | 3T | QSM, transcranial ultrasound | Voxel-wise analysis of the midbrain | PD: 23  HC: 27 | PD: 68.8 ± 8.7  HC: 65.7 ± 6.5 | PD: 8.5 ± 4.4 y | - Voxel clusters with significantly higher χ within the midbrain parenchyma, SNc, and rostral part of ventral tegmental area - No correlation of χ in any of the regions with MoCA | Medium |
| Thomas et al., (2020) | 3T | QSM | Voxel based analysis of whole GM,  ROIs: SN and substantia innominata | PD: 100  HC: 37 | PD: 64.5 ± 7.7  HC: 66.1 ± 9.4 | PD: 4.2 ± 2.5 y | - Higher χ in PD in the prefrontal cortex, rt. temporal cortex, rt. PUT, and SN - Higher χ in the HP, TH, and regions of ventromedial prefrontal cortex, basal forebrain and rostral CN correlated with lower MoCA scores. - Higher χ in the rt. PUT correlated with UPDRSIII. | Low |

| **Table S1c. Detailed summary of QSM studies in ALS** | | | | | | | | |
| --- | --- | --- | --- | --- | --- | --- | --- | --- |
| **Study** | **Field Strength** | **Modality** | **ROI** | **Sample size** | **Mean age ± SD (y)** | **Disease duration** | **Main findings** | **Quality assessment** |
| Schweitzer et al., (2015) | 3T | QSM, T2* | Relative motor cortex, right or left hand lobule | ALS: 12 PLS: 4 Control: 23 | MND: 56.3  HC: 56.6 | ALS: 20 m  PLS: 57.5 m | - Higher χ in the motor cortex in ALS and PLS patients compared to controls. - High sensitivity (87.5%) and specificity (87%) for QSM in diagnosis of MND from controls. - No significant difference in χ between ALS and PLS groups. | Medium |
| Costagli et al., (2016) | 7T | QSM. T2* | Cortical layers of M1 corresponding to Penfield's areas of the hand and foot | ALS: 17 HC: 13 | ALS: 62 ± 11  HC: 55 ± 11 | 17 ± 12 m | - In ALS patients, the deep layer of cortex corresponding to the most affected limb showed significantly increased χ compared to average χ in healthy controls. | Medium |
| Lee et al., (2017) | 3T | QSM | Hand lobule of the motor cortex and adjacent subcortical white matter. | ALS: 26 CVD: 26 HC: 26 | ALS: 61.08  CVD: 61.0  HC: 60.88 | ALS: 22.12 ± 21.38 m | - χ was higher in the motor cortex in ALS patients compared to CVD group and healthy controls, however, the comparison with HCs did not reach statistical significance. - In the subcortical white matter, ALS patients showed significantly lower χ compared to control group. - Maximum and mean χ in the left motor cortex inversely correlated with ALSFRS scores. However, for mean χ, this correlation did not reach a statistical significance. | Low |
| Acosta-Cabronero et al., (2018) | 3T | QSM, DTI | Whole-brain voxel-wise analysis  ROIs-based:  SN, SNc, CN, GP, PUT, TH, RN, DN, GP, AMY, HP, Precentral gyrus,  pars opercularis, corticospinal tract | ALS: 28 HC: 39 | ALS: 61  HC: 61 | ALS:18 ± 12 m | - In ALS patients: Higher χ in the motor cortex (precentral gyrus), SN, GP, RN, PUT, HP, and pars opercularis in the right side. - Lower χ in the corticospinal tract in ALS patients. - χ was higher in the SN and GP in bulbar-onset compared to limb-onset ALS patients. - There was no correlation between ALSFRS scores and χ in any of the investigated regions. - Correlation analysis between QSM and DTI showed positive correlation between χ and fractional anisotropy and negative correlation with mean diffusivity and radial diffusivity. | Low |
| Weidman et al., (2019) | 3T | DTI, QSM | Motor cortex | UMN: 43 (ALS: 38 /  PLS: 5)  Non-UMN controls: 15 | ALS: 60.6 ± 14.9 non-UMN: 58.5 ± 8.4 | ALS: 15.9 m non-UMN: 41.9 m | - Higher maximum χ in the motor cortex in ALS/PLS patients - DTI measures of the corticospinal tract were more accurate for differentiation of UMN group from controls. - Accuracy of maximum χ in the motor cortex for differentiation of UMN from control group was 0.632. | Medium |
| Welton et al., (2019) | 3T | QSM, Diffusion kurtosis imaging | Motor cortex | ALS: 21  HC: 63 | ALS: 54 ± 14 HC: 48 ± 18 | 1.8 ± 0.8 y | - Higher χ of the motor cortex in ALS patients - There was no correlation between χ and ALSFRS scores and disease duration. | Low |
| Donatelli et al., (2019) | 3T | QSM. T2* | Orofacial region of primary motor cortex | ALS-bulbar: 19  ALS: 36 | Whole cohort: 62 ± 9 | Whole cohort: 13.8 ± 10.3 m | - Majority of the ALS patients who had bulbar symptoms had T2* hypointensities in the orofacial primary motor cortex. - The group with T2* hypointensities showed significantly higher χ in QSM in the same region. | Medium |
| Contarino et al., (2020) | 3T | QSM | Motor cortex | ALS: 42  HC: 23 | ALS: ﻿61.4 ± 9.1  HC: ﻿57.4 ± 7.3 | ﻿27.5 ± 42.4 m | - The motor cortex in ALS patients showed higher mean χ but this difference did not reach statistical significance (p = 0.067) - Median χ value correlated with ALSFRS-R scores. - There was no correlation between χ and disease duration. | Low |

| **Table S1d. Detailed summary of QSM studies in WD** | | | | | | | | |
| --- | --- | --- | --- | --- | --- | --- | --- | --- |
| **Study** | **Field Strength** | **Modality** | **ROI** | **Sample size** | **Mean age ± SD (y)** | **Disease duration** | **Main findings** | **Quality assessment** |
| Fritzsch et al., (2014) | 7T | QSM, T2* | SN, RN, GP, head of CN, PUT | Neurologic WD: 6  Hepatic WD: 5  HC:10 | WD: 44  HC: 41 |  | - All WD patients compared to HC: increased χ in the bilateral SN, GP, RN and rt. PUT. - Neurologic WD: higher χ in the SN, GP, PT - Hepatic WD: higher χ in the SN, rt. GP, RN (lt. GP and rt. CN were borderline non-significant) | Low |
| Doganay et al., (2018) | 1.5T | QSM | GP, PUT, CN, TH, SN, posterior part of the pons | Neurologic WD: 11  HC: 14 | WD: 15 ± 3.3  HC: 13.2 ± 2.4 | WD: 5.27 y | - Higher χ in bilateral anterior and rt. posterior GP, bilateral posterior and rt. anterior PUT, rt. thalamus, rt. SN, and bilateral pons in patients with WD. | Medium |
| Saracoglu et al., (2018) | 1.5T | QSM | TH, brainstem, GP, PUT, head of CN, TH, SN, pons | Asymptomatic WD: 12  HC: 14 | Asymptomatic WD: 13.7 ± 3.3  HC: 13.2 ± 2.4 | Not applicable | - Increased χ in the pons, TH, and lt. posterior PUT in WD group. | Medium |
| Dezortova et al., (2019) | 3T | QSM | GP, PUT, CN, TH | 38 WD:  Neurologic WD: 28  Hepatic WD: 10  HC: 26 | Neurologic WD: 47.1 ± 9.5  Hepatic WD: 33.8 ± 10.9  HC: 44.8 ± 11.7 | Treatment duration:  Neurologic WD: ﻿17.6 ± 12.6 y  Hepatic WD: ﻿20.9 ± 11.4 y | - In all four regions, GP, PUT, CN, and TH, χ was higher in neurologic WD compared to both healthy controls and hepatic WD patients. | Low |

| **Table S1e. Detailed summary of QSM studies in HD** | | | | | | | | |
| --- | --- | --- | --- | --- | --- | --- | --- | --- |
| **Study** | **Field Strength** | **Modality** | **ROI** | **Sample size** | **Mean age ± SD (y)** | **Disease duration** | **Main findings** | **Quality assessment** |
| Domínguez et al., (2016) | 3T | QSM | GP, PUT, CN, TH | Premanifest HD: 31 Symptomatic HD: 32 HC: 30 | Pre-HD: 42.0 ± 8.9  Symptomatic-HD: 52.2 ± 9.2  HC: 40.4 ± 12.1 | Pre-HD, estimated years to diagnosis: 14.6 ± 5.9  Symptomatic-HD, since diagnosis: 1.9 ± 1.6 y | - Higher χ in the GP, PUT, and CN in both pre-HD and symptomatic-HD groups compared to HCs. - No difference in χ was observed in the TH. - χ in the PUT and CN was associated with disease burden score (﻿age×(CAG-35.5)). | Low |
| Van Bergen et al.,(2016) | 7T | QSM, R2* | CN, PUT, GP, AMY, HP, TH, SN, RN | Premanifest HD: 15 HC: 16 | premanifest HD: 42.4 ± 8.7  HC: 43.3 ± 11.7 | Not applicable | - Higher χ values in the CN, PUT and GP in premanifest-HD group. - Significant decreases in χ in the SN and HP in premanifest-HD. - χ values in the CN and PUT were found to be significantly correlated with atrophy and the CAG-age product scaled scores. - No correlation was found between χ in any of the ROIs and MoCA and UHDRS scores. | High (two different MRI acquisition protocols) |
| Chen et al., (2018) | 7T | QSM, R2* | CN, GP, SN,  HP, PT, RN | 24 HD gene mutation carriers: ﻿> 8 years pre‐HD: 9  ﻿<8 years pre‐HD: 6  early HD: 9 HC: 16 | ﻿> 8 years pre‐HD: ﻿34.4 ± 10.9  ﻿< 8 years pre‐HD: ﻿41.7 ± 11.5  early HD: ﻿50.8 ± 10.9 HC: ﻿44.8 ± 15.8 | Not reported/Not applicable | - Higher χ in the CN and PUT in closer-to-onset group compared to HC. - Higher χ in the CN, PUT, and GP in early HD group compared to HC, which significantly correlated with atrophy of these structures. - χ in the PUT and CN correlated with CAG-age product score. - There was no correlation between the measurements of motor, behavioural, and function (UHDRS) or MoCA scores and χ in any of the ROIs. - In close-to-onset and early-HD subjects, one-year follow up showed significant increase in χ of the CN and GP over this period. | Low |

| **Table S1f. Detailed summary of QSM studies in ataxic disorders** | | | | | | | | |
| --- | --- | --- | --- | --- | --- | --- | --- | --- |
| **Study** | **Field Strength** | **Modality** | **ROI** | **Sample size** | **Mean age ± SD (y)** | **Disease duration** | **Main findings** | **Quality assessment** |
| Harding et al., (2016) | 3T | QSM | DN, RN, SN, PUT, GP, TH, CN | FRDA: 30  HC: 33 | FRDA: 35.7 ± 12.2  HC: 36.9 ± 13.1 | 15.9 ± 7.7 y | - In the DN and RN, χ was higher in the FRDA group. This increased χ correlated with disease severity assessed by FARS, and longer GAA trinucleotide repeat in frataxin gene. - DN, SN, TH and RN showed significant atrophy in FRDA patients. | Medium |
| Ward et al., (2019) | 3T | Longitudinal QSM | DN | FRDA: 20  HC: 18 | FRDA: 34.3 ± 12.5  HC: 39.7 ± 13.3 | Not reported | - At both timepoints, higher χ was observed in bilateral DN in FRDA patients. - The increase in χ between two timepoints was significantly higher in FRDA patients, while rate of atrophy did not differ among groups. - There was a correlation between χ changes and disease severity or duration. | Low |
| Xie et al., (2019) | 3T | QSM | PUT, CN, GP, DN, SN, RN, Pons, motor cortex, motor white matter | SCA3: 18  HC: 18 | SCA3: 44.22 ± 5.51  HC: 41.33 ± 10.83 | Not reported | - In symptomatic SCA3 patients, χ was higher in the SN and RN compared to age-matched control group, while no significant difference in χ was found in any of the ROIs between preclinical SCA3 genetic carrier and age-matched healthy individuals. - No correlation was found between disease severity and χ in any of the regions | Medium |
| Sugiyama et al., (2019) | 3T | QSM | PUT, GP, CN, RN, SN, DN | MSA-C: 28  SCA6: 9  HC: 23 | MSA-C: 63.8 ± 9.5  SCA6: 60.7 ± 9.1  HC: 62.9 ± 8.1 | MSA-C: 3.9 ± 2.5  SCA6: 9.7 ± 5.9 years | - The MSA-C group showed higher χ in the DN compared to both HC and SCA6 groups. Further, χ in the SN was higher in MSA-C compared to healthy controls. - In MSA-C, χ of the DN showed significant correlation with disease duration, while no correlation was found between QSM and disease severity. - χ was found to be significantly lower in the DN in patients with SCA6 compared with healthy individuals. - In SCA6, DN χ inversely correlated with disease duration.   The AUC for DN χ was 0.925 in differentiation of SCA6 and MSA-C groups, and 0.834 in differentiation of MSA-C and HC groups. | Low |

| **Table S1g. Detailed summary of QSM studies in Myotonic dystrophy and Fabry disease** | | | | | | | | |
| --- | --- | --- | --- | --- | --- | --- | --- | --- |
| Study | Field Strength | Modality | ROI | Sample size | Mean age ± SD (y) | Disease duration | Main findings | Quality assessment |
| Ates et al. (2019) | 3T | QSM, R2* | TH, CN, PUT, GP, RN, SN, DN, HP, AMY, NA, STN | DM1: 12  DM2: 12  HC: 29 | DM1: 41 ± 13  DM2: 52 ± 6  HC: 44 ± 14 | DM1: 16 ± 7  DM2: 12 ± 6 | - χ was higher in the TH in DM1 compared to both DM2 and HC groups - DM2 patients showed higher χ in the PUT compared to healthy individuals. - In DM1, χ in the CN correlated with muscular impairment rating score and daytime sleepiness. - In DM2, χ in the CN correlated with depression score. | High (inconsistent MRI acquisition) |
| Russo et al. (2018) | 3T | QSM | Striatum (CN+PUT), GP, SN, RN, DN | Fabry disease: 30  HC: 37 | Fabry disease: 42.6 ± 12.2  HC: 43.2 ± 14.6 | Not reported | - In patients with Fabry disease, χ was higher in the SN and striatum compared to healthy individuals. - No correlation was found between QSM and clinical assessments. | Medium |

| **Table S1h. Detailed summary of QSM studies in NBIA syndromes** | | | | | | | | | | | |
| --- | --- | --- | --- | --- | --- | --- | --- | --- | --- | --- | --- |
| **Study** | **Field Strength** | **Modality** | **ROI** | | **Sample size** | **Mean age ± SD (y)** | | **Disease duration** | **Main findings** | | **Quality assessment** |
| Dusek et al., (2014) | 7T | QSM, SWI | GP, TH, PUT, CN, SN, RN, internal capsule | | PKAN: 2 heterozygous gene carriers: 11  HC: 13 | PKAN: 21 and 32 y/o  gene carriers: 43.4 ± 10.5  HC: 39.7 ± 13.6 | | Not reported | - No significant changes in χ was found in heterozygous PKAN2 mutation carriers compared to healthy controls. - In the two PKAN patients, χ was significantly increased in the GP (3-fold), SN (4-fold) and internal capsule (5-fold). | | Low |
| Zeng et al., (2019) | 3T | QSM | GP, TH, SN, RN, bilateral occipital subcortex | | PKAN: 6  HC: 8 | PKAN: 6-25 (range)  HC: 22.5 (mean) | | PKAN: 3-22 years (range) | - Higher χ in bilateral central GP, bilateral peripheral GP, and bilateral SN in patients with PKAN - χ was lower in the lt. RN in PKAN patients. | | Low |
| Dusek et al., (2019) | 7T | QSM, MRS | GP, SN, CN, PUT, TH | | MPAN: 4  C19orf12 mutation carriers: 9  HC: 19 | MPAN: 21.0 ± 2.9  C19orf12 mutation carriers: 50.4 ± 9.8  HC: 41.2 ± 14.9 | | Not reported | - Higher χ in the CN, GP, and SN in patients with MPAN compared to HC. - In patients with MPAN, volumes of the CN, PUT, and TH were significantly reduced compared to controls, while no difference in size was observed in mutation carriers in any ROI. - C19orf12 mutation heterozygous carriers had higher χ in the GP, SN, and TH. | | Low |
| χ: magnetic susceptibility  Aβ: Amyloid-beta  AD: Alzheimer’s disease  ALS: amyotrophic lateral sclerosis  ALSFRS: amyotrophic lateral sclerosis functional rating scale  aMCI: amnestic MCI  AMY: amygdala  AR: Akinetic-rigid  BOLD: Blood oxygen level dependent  CBD: cortical basal degeneration  CDRSOB: clinical dementia rating sum of boxes  CN: caudate nucleus  CVD: cerebrovascular disease  DKI: diffusion kurtosis imaging  DMN: default mode network  EOPD: Early-onset PD  ES-PD: Early stage PD  FARS: Friedreich Ataxia Rating Scale  FN: Fasciculata Nigrale  GM: grey matter  GP: globus pallidus | | | | GPe: Globus Pallidus externa  GPi: Globus Pallidus interna  GPl: lateral globus pallidus  GPm: medial globus pallidus  H&Y: Hoehn and Yahr scale  HAMA: Hamilton Anxiety Scale  HC: healthy control  HD: Huntington’s disease  HP: hippocampus  ILF: inferior longitudinal fasciculus  iRBD: idiopathic rapid eye movement sleep behaviour disorder  LEDD: levodopa equivalent dose  LS-PD: Late stage PD  lt.: left  M-LOPD: Middle-Late-onset PD  MADRS: Montgomery Asberg Depression Rating Scale  MCI: mild cognitive impairment  MMSE: Mini-mental state examination  MND: motor neuron disease | | | MoCA: Montreal cognition assessment  MPAN: Mitochondrial-membrane protein-associated neurodegeneration  MSA-C: MSA with predominant cerebellar ataxia  MSA-P: MSA with predominant parkinsonism  MSA: multiple system atrophy  NA: nucleus accumbens  NBIA: neurodegeneration with brain iron accumulation  NM: neuromelanin  NMS: non-motor symptoms  PD: Parkinson’s disease  PDQ-39: The Parkinson's Disease Questionnaire  PKAN: Pantothenate kinase-associated neurodegeneration  ppb: parts per billion  ppm: parts per million  PSP: progressive supranuclear palsy | | | PUL: pulvinar nucleus  PUT: putamen  RN: red nucleus  ROC: receiver operating characteristics  ROI: region of interest  rt.: right  SN: substantia nigra  SNc: substantia nigra pars compacta  SNr: substantia nigra pars reticulata  STN: subthalamic nucleus  SUVR: Standardized uptake value ratio  TD: Tremor-dominant  TH: thalamus  UHDRS: Unified Huntington’s disease rating scale  UMN: upper motor neuron  UPDRS: Unified Parkinson's Disease Rating Scale  VaD: vascular dementia  WD: Wilson’s disease | |
