# Supplementary material for: Systematic Review: Quantitative Susceptibility Mapping (QSM) of Brain Iron Profile in Neurodegenerative Diseases
Source: Front Neurosci. 2021 Feb 18;15:618435. doi: 10.3389/fnins.2021.618435 (PMC7930077; doi:10.3389/fnins.2021.618435)
Supplement: Supplementary Material 3 — Results and discussion sections on less studied neurodegenerative disorders. [file Table_3.DOCX]

**Supplementary material 3: Results and discussion sections on less studied neurodegenerative disorders**

**S3. Results**

**S3.1. Friedreich’s ataxia and spinocerebellar ataxia:**

Only two QSM studies that investigated FRDA and two QSM studies on SCA were identified and met criteria for inclusion in this review (Table S1f).

**S3.1.1. Magnetic susceptibility changes in FRDA**

The first QSM study investigating the differences of magnetic susceptibility in the subcortical brain regions in patients with FRDA and healthy individuals showed significantly higher susceptibility in the DN and RN in the FRDA group (Table 2i). Other subcortical structures did not show any variation in χ compared to the control group. Magnetic susceptibility in the DN correlated with disease severity scores and length of GAA trinucleotide repeats (Harding et al., 2016). In a longitudinal study by the same team, higher susceptibility in the DN at both time points was confirmed, with a significantly higher rate of increase in susceptibility between the two time-points found in patients with FRDA. Interestingly, although the volume of the DN was lower in FRDA group at both time-points, rate of atrophy did not significantly vary among groups (Ward et al., 2019).

**S3.1.2. Magnetic susceptibility changes in SCA**

In a study by Xie et al. (2019) in eighteen patients with SCA3, higher susceptibility was observed in the substantia nigra, RN, and GP compared to healthy individuals, however, the difference did not reach statistical significance in the GP (p-value= 0.056). None of the other ROIs including the DN, CN, putamen, and pons showed any difference between groups. The group of twelve asymptomatic pre-SCA3 subjects did not show any changes in susceptibility in any of the subcortical or cortical ROIs compared to healthy controls (Table 2i). Furthermore, increased regional susceptibility in patients with SCA3 did not correlate with ataxia severity scales. In another QSM study of brain iron distribution in subcortical structures (putamen, GP, CN, RN, substantia nigra, and DN) of nine patients with SCA6 compared to healthy participants, a significantly lower susceptibility was observed in the DN, which inversely correlated with disease duration (Sugiyama et al., 2019) (Table S1f).

**S3.2. Fabry disease**

QSM was used in one study in Fabry disease (FD), which compared 30 patients with FD with 37 age- and sex-matched healthy controls. In this study there was significantly increased susceptibility in the SN and striatum in FD (p<0.001) but no differences in the GP, RN and DN. Increased susceptibility correlated with the atrophy of the SN, but there were no associations with the clinical severity measures, such as UPDRS, Scale of Assessment and Rating of Ataxia and Timed Up and Go Test (Table S1g).

**S3.3. Myotonic Dystrophy**

**S3.3.1. Magnetic susceptibility changes in DM in comparison with healthy individuals**

Only one study on type 1 myotonic dystrophy (DM1) and type 2 myotonic dystrophy (DM2) was included that used QSM and R2* imaging (Ates et al., 2019). QSM showed significantly higher susceptibility in the thalamus in DM1 compared to DM2 and control groups. In patients with DM2, susceptibility was higher in the putamen compared to healthy individuals. DM1 patients showed significant atrophy in the putamen, nucleus accumbens, thalamus, and CN while in the DM2 group, only the nucleus accumbens had significantly reduced volume compared to healthy controls. QSM was not correlated with atrophy in any of the regions in either subtypes of DM (Ates et al., 2019) (Table S1g).

**S3.3.2. Correlation of QSM findings in DM with clinical features and other biomarkers**

In patients with DM1, severity of motor impairments and daytime sleepiness was associated with increased susceptibility in the CN, while impaired flexibility of thinking correlated with susceptibility in the hippocampus. In none of the studied ROIs, a correlation between susceptibility and CTG trinucleotide repeat length was found. In patients with DM2, there was a correlation between increased susceptibility in CN with depression, in SN with parkinsonian symptoms, and in thalamus with impaired tonic alertness (Ates et al., 2019).

**S3.4. Neurodegeneration with brain iron accumulation syndromes (NBIA)**

Pantothenate-kinase-associated neurodegeneration (PKAN) is one of the NBIA syndromes, with autosomal recessive transmission. Two studies investigated iron distribution in PKAN using QSM. In the study by Dusek et al. (2014) two patients with PKAN, 11 asymptomatic subjects with heterozygous mutation of the pantothenate kinase-2 gene, and 13 healthy individuals were compared using QSM in different subcortical ROIs. The two patients with PKAN showed significantly higher susceptibility in the GP (3-fold increase), SN (4-fold increase) and internal capsule (5-fold increase), while in the genetic carriers, there was no difference of susceptibility in any of the regions compared to healthy subjects (Dusek et al., 2014). In a later study with a larger population of patients with PKAN (n=6), Zeng et al. (2019) confirmed the increased susceptibility in the GP and SN, while in the left RN, the patient group showed lower susceptibility compared to controls.

One QSM study examined mitochondrial membrane protein-associated neurodegeneration (MPAN), another syndrome of NBIA. As expected, significantly higher susceptibility was found in the SN, GP, and CN in the four patients with MPAN compared to healthy controls. In contrast to PKAN, where heterozygous carriers did not show any increased iron burden in the brain, the asymptomatic heterozygous *c19orf12* gene carriers were found to have increased susceptibility in the CN and putamen, but not in the other regions (Dusek et al., 2019) (see Tables 2j and S1h ).

**S4. Discussion**

**S4.1. Friedreich’s ataxia and spinocerebellar ataxia**

FRDA is an autosomal recessive genetic disorder caused by GAA trinucleotide repeat expansion in frataxin gene. Frataxin is a mitochondrial protein that binds iron and promotes incorporation of iron into iron-sulphur clusters, essential for energy production in the mitochondria. Without the proper function of frataxin, inadequate energy production and deposition of excess iron ensues (Chiang et al., 2016). FRDA is characterized by mixed sensory and cerebellar ataxia and motor dysfunction (Koeppen, 2011; Llorens et al., 2019). The main brain region involved in neurodegeneration and atrophy in FRDA is the DN, an iron-rich nucleus within the cerebellum. Neuroimaging and histopathological studies have shown significant atrophy and neuronal loss in the DN (Selvadurai et al., 2018), with two QSM studies on FRDA reporting higher susceptibility in the DN. Although this finding on QSM likely due to increased iron, results from histological examinations have been inconsistent in this regard. While some histological studies have reported increased iron in the DN, others have found no difference between FRDA patients and controls (Llorens et al., 2019).

SCA is an umbrella term referring to a group of mostly autosomal dominant genetic disorders, all of which have progressive ataxia as a common feature. To date, more than 40 subtypes of SCA have been identified based on the causative genetic locus. In most cases of SCA, the disease results from trinucleotide (CAG) repeat expansions, similar to HD. Neural damage and atrophy of the cerebellum is the most common feature among SCAs, however, each subtype shows a specific pattern of involvement in the brain and clinical manifestations (Klockgether et al., 2019).

Two QSM studies report findings in SCA3 and SCA6 subtypes. SCA3 is the most common type of SCA, neuropathologically characterised by neural loss in the DN and basal ganglia (Klockgether et al., 2019). A QSM study on SCA3 detected increased susceptibility in the SN and RN in 18 patients with SCA3, consistent with reports of involvement of these regions. Mean susceptibility in the DN was higher in SCA3 but did not reach statistical significance.

SCA6 is the third most common type of SCA after SCA3 and SCA2. Unlike SCA3, SCA6 is characterised by neural cell loss is in the cerebellar cortex rather than the DN (Klockgether et al., 2019). Only one QSM study investigated a small group of patients with SCA6 (n=9) and reported decreased susceptibility in the DN. Lower susceptibility can be explained by either the removal of paramagnetic substances (iron) or build-up of diamagnetic materials such as myelin or calcium. Considerable atrophy of the cerebellar cortex with mostly preserved volume of DN has been reported in SCA6 (Deistung et al., 2016). It is possible that redistribution of iron within the cerebellum is the reason behind lower susceptibility in the DN. Calcification and migration of astrocytes that contain the lowest amounts of iron, as reported by previous studies, are other possible explanations for this finding (Knight et al., 2004; Sugiyama et al., 2019). Considering the small number of QSM studies in SCA and FRDA, future studies would provide a clearer picture of the complex pathologies underlying these ataxic disorders.

**S4.2. Fabry disease**

Fabry disease (FD) is an X-linked disorder characterised by progressive deposition of globotriaosylceramide caused by the deficiency of alpha-galactosidase A lysosomal enzyme. The most common CNS pathology observed in FD are micro- and macrovascular events such as cerebral microbleeds, ischemic stroke, which result from deposition of globotriaosylceramide in the endothelium and smooth muscles of the vessel walls. MRI findings are nonspecific and include T_1_ hyperintensities in the white matter and pulvinar nucleus (pulvinar sign). Limited evidence suggests reduction of intracranial volume and regional atrophy in the hippocampus and thalamus (Cocozza et al., 2018).

Currently, there is no evidence for the association of iron in this disease. The only one QSM study in FD has identified higher susceptibility in the striatum and SN (Russo et al., 2018). These findings may be associated with the motor impairments observed in FD (Löhle et al., 2015). For interpretation of susceptibility changes in FD, it is important to also keep in mind about the vasculopathy and cerebrovascular events, and the possibility of iron deposition resulting from microhemorrhages. Future studies would provide additional insights into the QSM changes in FD.

**S4.3. Myotonic dystrophy**

DMs are multi-system autosomal dominant genetic disorders characterised by muscular dystrophy (weakness and wasting of the muscles), myotonia (inability to relax contracted voluntary muscles), cardiac conductive defects and endocrine disorders (Meola & Cardani, 2015). While muscles are primarily affected, widespread pathologic changes in the brain lead to a variety of neuropsychiatric manifestations including intellectual disability, cognitive deficits, and sleep disturbances (Wenninger et al., 2018). Structural changes in the brain, which are more prevalent in DM1, include white matter lesions (predominantly in the frontal and temporal lobes) and atrophy of the cortical grey matter, subcortical grey matter, and corpus callosum (Minnerop et al., 2018).

A single QSM study of DM found increased susceptibility in the thalamus in DM1 and in the putamen in DM2, while abnormal R_2_^*^ signal was detected in widespread subcortical regions including the CN, putamen and nucleus accumbens in both DM1 and DM2 groups and GP, hippocampus, SN and STN in patients with DM1 compared to healthy individuals. The inconsistency between the findings of R_2_^*^ and QSM in this study makes it difficult to determine the extent to which these changes could be attributed to iron load. There are no post-mortem reports of brain iron changes in DM, although studies have identified reduced production of ferritin heavy chain as a result of CTG repeat expansions in DM (Watanabe et al., 1999).

**S4.4. Syndromes of neurodegeneration with brain iron accumulation (NBIA)**

NBIA syndromes are rare genetic neurodegenerative disorders characterised by excessive iron deposition in the basal ganglia, especially the GP. The clinical hallmark of these syndromes is progressive hypo- and/or hyper-kinetic abnormal movements (Hogarth, 2015). To date, 10 distinct NBIA syndromes have been identified. Pantothenate-kinase-associated neurodegeneration (PKAN) is the most common of NBIA syndromes (about half of the cases), which is caused by a mutation in pantothenate kinase-2 gene. The main neuroimaging finding in PKAN is the “eye of the tiger” sign in the GP, characterized by a central hyperintense region surrounded by a hypointense area on T2-weighted MRI (Hayflick et al., 2018). Two studies investigating iron distribution in PKAN using QSM have identified markedly increased susceptibility (3-4-fold) in the GP and SN compared to controls. One of the studies reported decreased susceptibility in the left RN, which may be explained by the small sample and the segmentation method used.

Mitochondrial membrane-protein associated neurodegeneration (MPAN) is another NBIA syndrome, caused by a mutation in the *C19orf12* gene, leading to neuropathy, cognitive impairment, optic nerve atrophy and movement disorder. T2-weighted imaging reports show hypointense signals in the GP and SN (Hayflick et al., 2018). Similarly, the one available QSM study reported increased susceptibility in the GP, SN as well as CN (Dusek et al., 2019). Although MPAN follows an autosomal recessive inheritance pattern, symptoms have been reported in those with one mutated allele (Hayflick et al., 2018). Reflecting the brain involvement in these individuals, QSM has shown increased susceptibility in the CN and putamen even in asymptomatic heterozygous carriers (Dusek et al., 2019).

**References:**

Ates, S., Deistung, A., Schneider, R., Prehn, C., Lukas, C., Reichenbach, J. R., Schneider-Gold, C., & Bellenberg, B. (2019). Characterization of Iron Accumulation in Deep Gray Matter in Myotonic Dystrophy Type 1 and 2 Using Quantitative Susceptibility Mapping and R2* Relaxometry: A Magnetic Resonance Imaging Study at 3 Tesla. *Frontiers in Neurology*, *10*, 1320. https://doi.org/10.3389/fneur.2019.01320

Chiang, S., Kovacevic, Z., Sahni, S., Lane, D. J. R., Merlot, A. M., Kalinowski, D. S., Huang, M. L.-H., & Richardson, D. R. (2016). Frataxin and the molecular mechanism of mitochondrial iron-loading in Friedreich’s ataxia. In *Clinical Science* (Vol. 130, Issue 11, pp. 853–870). Clin Sci (Lond). https://doi.org/10.1042/CS20160072

Cocozza, S., Russo, C., Pontillo, G., Pisani, A., & Brunetti, A. (2018). Neuroimaging in Fabry disease: current knowledge and future directions. In *Insights into Imaging* (Vol. 9, Issue 6, pp. 1077–1088). Springer. https://doi.org/10.1007/s13244-018-0664-8

Deistung, A., Stefanescu, M. R., Ernst, T. M., Schlamann, M., Ladd, M. E., Reichenbach, J. R., & Timmann, D. (2016). Structural and Functional Magnetic Resonance Imaging of the Cerebellum: Considerations for Assessing Cerebellar Ataxias. In *Cerebellum* (Vol. 15, Issue 1, pp. 21–25). Springer. https://doi.org/10.1007/s12311-015-0738-9

Dusek, P., Mekle, R., Skowronska, M., Acosta-Cabronero, J., Huelnhagen, T., Robinson, S. D., Schubert, F., Deschauer, M., Els, A., Ittermann, B., Schottmann, G., Madai, V. I., Paul, F., Klopstock, T., Kmiec, T., Niendorf, T., Wuerfel, J., & Schneider, S. A. (2019). Brain iron and metabolic abnormalities in C19orf12 mutation carriers: A 7.0 tesla MRI study in mitochondrial membrane protein–associated neurodegeneration. *Movement Disorders*, mds.27827. https://doi.org/10.1002/mds.27827

Dusek, P., Tovar Martinez, E. M., Madai, V. I., Jech, R., Sobesky, J., Paul, F., Niendorf, T., Wuerfel, J., & Schneider, S. A. (2014). 7-Tesla Magnetic Resonance Imaging for Brain Iron Quantification in Homozygous and Heterozygous PANK2 Mutation Carriers. *Movement Disorders Clinical Practice*, *1*(4), 329–335. https://doi.org/10.1002/mdc3.12080

Harding, I. H., Raniga, P., Delatycki, M. B., Stagnitti, M. R., Corben, L. A., Storey, E., Georgiou-Karistianis, N., & Egan, G. F. (2016). Tissue atrophy and elevated iron concentration in the extrapyramidal motor system in Friedreich ataxia: the IMAGE-FRDA study. *Journal of Neurology, Neurosurgery & Psychiatry*, *87*(11), 1261–1263. https://doi.org/10.1136/jnnp-2015-312665

Hayflick, S. J., Kurian, M. A., & Hogarth, P. (2018). Neurodegeneration with brain iron accumulation. In *Handbook of Clinical Neurology* (Vol. 147, pp. 293–305). Elsevier. https://doi.org/10.1016/B978-0-444-63233-3.00019-1

Hogarth, P. (2015). Neurodegeneration with Brain Iron Accumulation: Diagnosis and Management. *Journal of Movement Disorders*, *8*(1), 1–13. https://doi.org/10.14802/jmd.14034

Klockgether, T., Mariotti, C., & Paulson, H. L. (2019). Spinocerebellar ataxia. *Nature Reviews Disease Primers*, *5*(1), 24. https://doi.org/10.1038/s41572-019-0074-3

Knight, M. A., Gardner, R. J. M. K., Bahlo, M., Matsuura, T., Dixon, J. A., Forrest, S. M., & Storey, E. (2004). Dominantly inherited ataxia and dysphonia with dentate calcification: Spinocerebellar ataxia type 20. In *Brain* (Vol. 127, Issue 5, pp. 1172–1181). Oxford Academic. https://doi.org/10.1093/brain/awh139

Koeppen, A. H. (2011). Friedreich’s ataxia: Pathology, pathogenesis, and molecular genetics. In *Journal of the Neurological Sciences* (Vol. 303, Issues 1–2, pp. 1–12). NIH Public Access. https://doi.org/10.1016/j.jns.2011.01.010

Llorens, J. V., Soriano, S., Calap-Quintana, P., Gonzalez-Cabo, P., & Moltó, M. D. (2019). The role of iron in Friedreich’s ataxia: Insights from studies in human tissues and cellular and animal models. In *Frontiers in Neuroscience* (Vol. 13, Issue FEB, p. 75). Frontiers. https://doi.org/10.3389/fnins.2019.00075

Löhle, M., Hughes, D., Milligan, A., Richfield, L., Reichmann, H., Mehta, A., & Schapira, A. H. V. (2015). Clinical prodromes of neurodegeneration in Anderson-Fabry disease. *Neurology*, *84*(14), 1454–1464. https://doi.org/10.1212/WNL.0000000000001450

Meola, G., & Cardani, R. (2015). Myotonic dystrophies: An update on clinical aspects, genetic, pathology, and molecular pathomechanisms. *Biochimica et Biophysica Acta (BBA) - Molecular Basis of Disease*, *1852*(4), 594–606. https://doi.org/10.1016/J.BBADIS.2014.05.019

Minnerop, M., Gliem, C., & Kornblum, C. (2018). Current progress in CNS imaging of myotonic dystrophy. In *Frontiers in Neurology* (Vol. 9, Issue AUG, p. 646). Frontiers Media SA. https://doi.org/10.3389/fneur.2018.00646

Russo, C., Pontillo, G., Pisani, A., Saccà, F., Riccio, E., Macera, A., Rusconi, G., Stanzione, A., Borrelli, P., Brescia Morra, V., Tedeschi, E., Brunetti, A., Cocozza, S., & Palma, G. (2018). Striatonigral involvement in Fabry Disease: A quantitative and volumetric Magnetic Resonance Imaging study. *Parkinsonism and Related Disorders*, *57*, 27–32. https://doi.org/10.1016/j.parkreldis.2018.07.011

Selvadurai, L. P., Harding, I. H., Corben, L. A., & Georgiou-Karistianis, N. (2018). Cerebral abnormalities in Friedreich ataxia: A review. *Neuroscience and Biobehavioral Reviews*, *84*(March 2017), 394–406. https://doi.org/10.1016/j.neubiorev.2017.08.006

Sugiyama, A., Sato, N., Kimura, Y., Fujii, H., Maikusa, N., Shigemoto, Y., Suzuki, F., Morimoto, E., Koide, K., Takahashi, Y., Matsuda, H., & Kuwabara, S. (2019). Quantifying iron deposition in the cerebellar subtype of multiple system atrophy and spinocerebellar ataxia type 6 by quantitative susceptibility mapping. *Journal of the Neurological Sciences*, *407*, 116525. https://doi.org/10.1016/j.jns.2019.116525

Ward, P. G. D., Harding, I. H., Close, T. G., Corben, L. A., Delatycki, M. B., Storey, E., Georgiou-Karistianis, N., & Egan, G. F. (2019). Longitudinal evaluation of iron concentration and atrophy in the dentate nuclei in friedreich ataxia. *Movement Disorders*, *34*(3), 335–343. https://doi.org/10.1002/mds.27606

Watanabe, T., Sasagawa, N., Usuki, F., Koike, H., Saitoh, N., Sorimachi, H., Maruyama, K., Nakase, H., Takagi, A., Ishiura, S., & Suzuki, K. (1999). Overexpression of myotonic dystrophy protein kinase in C2C12 myogenic culture involved in the expression of ferritin heavy chain and interleukin-1α mRNAs. *Journal of the Neurological Sciences*, *167*(1), 26–33. https://doi.org/10.1016/S0022-510X(99)00133-1

Wenninger, S., Montagnese, F., & Schoser, B. (2018). Core clinical phenotypes in Myotonic Dystrophies. In *Frontiers in Neurology* (Vol. 9, Issue MAY, p. 303). Frontiers Media SA. https://doi.org/10.3389/fneur.2018.00303

Xie, F., Weihua, L., Lirong, O., Wang, X., & Xing, W. (2019). Quantitative susceptibility mapping in spinocerebellar ataxia type 3/Machado–Joseph disease (SCA3/MJD). *Acta Radiologica*, 028418511986890. https://doi.org/10.1177/0284185119868906

Zeng, J., Xing, W., Liao, W., & Wang, X. (2019). Magnetic resonance imaging, susceptibility weighted imaging and quantitative susceptibility mapping findings of pantothenate kinase-associated neurodegeneration. *Journal of Clinical Neuroscience*, *59*, 20–28. https://doi.org/10.1016/j.jocn.2018.10.090
