# Supplementary material for: Systematic Review: Quantitative Susceptibility Mapping (QSM) of Brain Iron Profile in Neurodegenerative Diseases
Source: Front Neurosci. 2021 Feb 18;15:618435. doi: 10.3389/fnins.2021.618435 (PMC7930077; doi:10.3389/fnins.2021.618435)
Supplement: Supplementary Material 4 — Table S2. Details of MRI acquisition and QSM processing methods used by reviewed studies. [file Table_4.DOCX]

| **Table S2. Details of MRI acquisition and QSM processing methods used by reviewed studies** | | | | | | | | | | | | |
| --- | --- | --- | --- | --- | --- | --- | --- | --- | --- | --- | --- | --- |
| **Study** | **Disease** | **Field strength** | **vox size (mm)** | **TE (ms)** | **∆TE (ms)** | **number of echoes** | **Acquisition sequence** | **Phase unwrapping** | **Background field removal** | **Dipole inversion** | **Reference region** | **Further description provided for QSM construction** |
| Acosta-Cabronero et al., (2013) | AD | 3T | 1×1×2 | 20 |  |  | Fully-flow compensated, radio-frequency-spoiled 3D fast low-angle shot (FLASH) | Laplacian-based |  | MEDI | homogeneous bilateral posterior ventricular region |  |
| Van Bergen et al., (2016) | AD | 7T | 0.5×0.5×0.5 | 6 | 6 | 3 (2 echoes used) | GRE | Laplacian-based | V-SHARP | LSQR | frontal central CSF |  |
| Moon et al., (2016) | AD | 3T | 0.94×0.94×2 | 3.5 | 4.09 | 8 | GRE (based on SWAN) | Magnitude-guided | PDF | MEDI |  | MEDI toolbox  (J. Liu et al., 2012; T. Liu et al., 2011) |
| Hwang et al., (2016) | AD | 3T | 0.63×0.63×1.26 | 34 | NA | 1 | GRE (first-order flow-compensated 3D gradient-echo sequence) | Quality-guided | PDF | MEDI | posterior part of a ventricle |  |
| Ayton et al., (2017) | AD | 3T | 0.93×0.93×1.75 | 20 |  |  | GRE | Laplacian-based | V-SHARP | iLSQR | middle-frontal white matter region |  |
| Kim et al., (2017) | AD | 3T | 0.68×0.68×2.2 | 3.4 | 6 | 7 | 3D fast field-echo (FFE) |  | PDF | MEDI | bilateral posterior ventricular region |  |
| L. Du et al., (2018) | AD | 3T | 1×1×1 | 3.2 |  |  | GRE |  |  |  |  |  |
| Tiepolt et al., (2018) | AD | 7T | O.7×0.7×0.7 | 10 |  |  | GRE | SDI QSM processing based on (Schweser et al., 2013) | | | CSF |  |
| Van Bergen et al., (2018) | AD | 3T | 1×1×1 | 6 | 4 | 6 | bipolar GRE | Laplacian-based | SHARP | iLSQR | deep frontal white matter |  |
| Meineke et al., (2018) | AD | 3T | 0.6×0.6×2 | 3.5 | 4 | 7 | GRE | QSM processing based on JEDI algorithm | | | corpus callosum |  |
| Kan et al., (2020) | AD | 3T | 1×1×1 | 6 | 6.2 | 5 | monopolar MP-QSM | Laplacian-based | V-SHARP | iLSQR | CSF |  |
| Kagerer et al., (2020) | AD | 3T | 1×1×1× | 6 | 4 | 6, last 3 echoes used | GRE | Laplacian-based | V-SHARP | LSQR | deep frontal white matter |  |
| Tuzzi et al., (2020) | AD | 9.4 T | 0.13×0.13×0.61 | 16.5 | NA | 1 | GRE | Laplacian-based | RE-SHARP | iLSQR | whole brain |  |
| Lotfipour et al., (2012) | PD | 7T | 0.67×0.67×0.70 | 25 |  |  | fast field echo segmented echo planar imaging (EPI factor 3) | FSL Prelude |  |  | white matter posterior to the red nuclei and medial to the posterior portions of the SN |  |
|  | PD |  | 0.4×0.4×0.5 0.5×0.5×0.5 | 20 |  |  | fast field echo sequence (EPI factor 3) |  |  |  |  |  |
| Ide et al., (2014) | PD | 3T | 0.69×0.4×1.5 | 4.5 | 5 | 11 | GRE | MEDI, based on (De Rochefort et al., 2010; J. Liu et al., 2012) | | |  |  |
| Barbosa et al., (2015) | PD | 3T | 0.48×0.48×2 | 7.7 | 12 | 4 | GRE | FSL Prelude | TSVD-SHARP | TKD | occipital white matter |  |
| He et al., (2015) | PD | 3T | 0.47×0.47×2 | 5.5 | 6.41 | 8 | GRE |  | SHARP | iLSQR | whole brain average |  |
| Murakami et al., (2015) | PD | 3T | 0.69×0.4×1.5 | 4.5 | 5 | 11 | GRE | Magnitude-guided | PDF | MEDI |  |  |
| Azuma et al., (2016) | PD | 3T | 0.9×0.9×2 | 6.2 | 6.2 | 8 | GRE | morphology-enabled dipole inversion method based on (Graham et al., 2000; Schweser et al., 2012) | | |  |  |
| Peckham et al., (2016) | PD | 3T | 1×0.5×2 | 20 |  |  | axial 3D SWI | SPIN (Signal Processing In Nuclear magnetic resonance) processing pipeline (Haacke et al., 2010; Tang et al., 2013). | | |  |  |
| Langkammer et al., (2016) | PD | 3T | 0.9×0.9×2 | 4.92 | 4.92 | 6 - even echoes used | GRE |  | V-SHARP | HEIDI | CSF |  |
| Du et al., (2016) | PD | 3T | 0.9×0.9×2 | 6.2 | 6.2 | 8 | GRE | morphology-enabled dipole inversion (MEDI) with nonlinear formulation method (T. Liu et al., 2012, 2013) | | |  |  |
| He et al., (2017) | PD | 3T | 0.86×0.86×1 | 2.7 | 2.9 | 16 | GRE |  | SHARP | iLSQR | whole brain average |  |
| Ito et al., (2017) | PD | 3T | 0.5×1.14×2 | 15 | NA | 1 | GRE |  | RE-SHARP | MUDICK | internal capsule and corpus callosum |  |
| Sjöström et al., (2017) | PD | 3T | 0.7×0.7×1.6 | 20 | NA | 1 | GRE | Laplacian-based | V-SHARP | iLSQR | CSF lateral ventricles | STI Suite software |
|  | PD | 1.5T | 0.9×0.9×2 | 40 | NA | 1 |  |  |  |  |  |  |
|  | PD | 1.5T | 0.9×0.9×1.6 | 40 | NA | 1 |  |  |  |  |  |  |
| Xuan et al., (2017) | PD | 3T | Slice thickness:2.8 | 5 | 5.02 | 8 | GRE | Laplacian-based | V-SHARP | iLSQR |  | STI Suite software |
| Guan et al., (2017b) | PD | 3T | 0.78×0.78×2.8 | 5 | 5.02 | 8 | GRE (ESWAN) | Laplacian-based | V-SHARP | iLSQR |  | STI Suite software |
| Guan et al., (2017a) | PD | 3T | 0.78×0.78×2.8 | 5 | 5.02 | 8 | GRE (flow-compensated, 3D-ESWAN) | Laplacian-based | V-SHARP | iLSQR | CSF | STI Suite software |
| Zhao et al., (2017) | PD | 3T | 0.62×0.62×2 | 6.8 | 6.8 | 8 | GRE |  | PDF | MEDI | occipital white matter | Based on (J. Liu et al., 2012) |
| Acosta-Cabronero et al., (2017) | PD | 3T | 1×1×2 | 20 | NA | 1 | GRE | Laplacian-based | SHARP | nMEDI | not referenced |  |
| Takahashi et al., (2018) | PD | 3T | 0.57×0.85×2.4 | 13 | 4.72 | 6 | GRE | MEDI (T. Liu et al., 2012) | | |  |  |
| Kim et al., (2018) | PD | 3T | 0.5×0.5×1 | 11.1 | 11.1 | 6 | GRE (Oblique coronal 3D multiecho data image combination (MEDIC)) | STI Suite software package | | | decussation of the superior cerebellar peduncle on each resliced QSM images |  |
| Du et al., (2018) | PD | 3T | 0.9×0.9×2 | 6.2 | 6.2 | 8 | GRE | MEDI, nonlinear formulation | | |  |  |
| Shin et al., (2018) | PD | 3T | 0.78×0.78×2 | 25.7-28.5 | NA | 1 | GRE | a susceptibility tensor imaging software suite (version 2.2, Updated on Jan. 8, 2014; Brain Imaging & Analysis Center, Durham, NC, USA) (Li, Avram, et al., 2014; Li, Wu, et al., 2014) | | |  |  |
| Takahashi et al., (2018) | PD | 3T | 0.57×0.86×2.4 | 36.4 |  |  | GRE | MEDI (T. Liu et al., 2012) | | |  |  |
| An et al., (2018) | PD | 3T | 0.62×0.62×2 | 6.8 |  | 8 | GRE | one-dimensional temporal unwrapping | PDF | MEDI |  |  |
| Li et al., (2018) | PD | 3T | 0.9×0.9×1 | 23 | NA | 1 | SWI (velocity-compensated 3D fast-field echo sequence) | Laplacian-based | RE-SHARP | L1-norm total-variation- based regularization algorithm with magnitude image as structural prior | CSF of the lateral ventricle |  |
| Mazzucchi et al., (2019) | PD | 3T | 0.93×0.93×1 | 13 | 3.3 | 16 | GRE (SWAN) | Laplacian-based | V-SHARP | iLSQR | subcortical white matter of the right occipital lobe |  |
| Miyata et al., (2019) | PD | 3T | 0.69×0.39×1.5 | 4.5 | 5 | 11 | GRE | (MEDI) technique, (J. Liu et al., 2012; Wallace et al., 2016) | | |  |  |
| Guan et al., (2019a) | PD | 3T | 0.78×0.78×2.8 | 5 | 5.02 | 8 | GRE (ESWAN) | Laplacian-based | V-SHARP | iLSQR | CSF | STI suite |
| Li et al., (2019) | PD | 3T | 0.62×0.62×2 | 6.8 | 6.8 | 8 | GRE | Magnitude-guided | PDF | MEDI | occipital white matter |  |
| Azuma et al., (2019) | PD | 3T | 0.9×0.9×2 | 6.2 | 6.2 | 8 | GRE | MEDI (Wang & Liu, 2015) | | |  |  |
| Guan et al., (2019b) | PD | 3T | 0.58×0.62×2 | 4.55 | 3.65 | 8 | GRE (ESWAN) | Laplacian-based | V-SHARP | STAR-QSM | whole brain average | STI suite |
| Shahmaei et al., (2019) | PD | 3T | 1×1×1.5 | 4 |  |  | GRE |  | SHARP |  |  |  |
| Sethi et al., (2019) | PD | PD: 3T | 0.5×0.5×2 | 6 and 20, second echo used | | 1 | SWI |  |  | an iterative thresholded k-space division | | an in-house Matlab toolbox (SMART, The MRI Institute for Biomedical Research, Detroit, MI) |
|  |  | HC: 1.5T | 0.6×0.75×3 | 40 | NA | 1 | GRE |  |  |  |  |  |
| Uchida et al., (2019) | PD | 3T | 1×1×1 | 6.4 | 6.4 | 5 | 3D fast low angle shot sequence | Laplacian-based | V-SHARP | iLSQR |  |  |
| Sun et al., (2019) | PD | 3T | 0.67×0.67×1.5 | 17.5 | NA | 1 | GRE | Laplacian-based | V-SHARP | STAR-QSM | bilateral posterior limb of the internal capsule | STI Suite software |
| Cheng et al., (2019) | PD | 3T | 0.86×0.86×1 | 2.7 | 2.9 | 16 | GRE |  | SHARP | iLSQR |  | method based on (Li et al., 2011) |
| Ghassaban et al., (2019) | PD | 3T | 0.86×0.86×1 | 2.69 | 2.87 | 16, first 8 echoes used | GRE | quality guided 3D phase unwrapping algorithm (3DSRNCP) | SHARP | TKD |  | SMART v2.0 (MRI Institute for Biomedical Research, Bingham Farms, MI, United States) |
| Wang et al., (2019) | PD | 3T | 1×1×1.5 | 7 | 8 | 6 | GRE | MEDI with a nonlinear formulation (T. Liu et al., 2012, 2013) | | |  |  |
| Bergsland et al., (2019) | PD | 3T | 0.5×1×2 | 22 | NA | 1 | GRE | best-path algorithm | V-SHARP | HEIDI | whole brain average |  |
| Hwang et al., (2019) | PD | 3T | 0.72×0.72×2 | 4.92 | 2.46 | 7 | 3D GE | Laplacian-based | V-SHARP | iLSQR |  | STI Suite |
| Chen et al., (2019) | PD | 3T | 0.86×0.69×2 | 20 |  |  | GRE | quality guided 3D phase unwrapping algorithm (3DSRNCP) | SHARP |  |  | SMART 2.0 (MRI Institute for Biomedical Research, Detroit, MI) (M. Liu et al., 2016) |
| Cheng et al., (2020) | PD | 3T | 0.67×0.67×1.34 | 11 | 9 | 2 | GRE | iterative susceptibility weighted imaging and mapping (iSWIM) (Tang et al., 2013), using SPIN software (SpinTech, Inc., Bingham Farms, MI, USA). | | |  |  |
| Ahmadi et al., (2020) | PD | 3T | 1×1×1 | 10 | 10 | 3 | GRE | a fast QSM technique based on a total-generalized-variation (TGV) approach (Langkammer et al., 2015) | | |  |  |
| Thomas et al., (2020) | PD | 3T | 1×1×1 | 18 | NA | 1 | GRE | Laplacian-based | MSDI | |  | QSMbox (https://gitlab.com/acostaj/QSMbox) pipeline |
| Schweitzer et al., (2015) | ALS/PLS | 3T | 0.57×0.75×2 | 5 | 5 | 11 | GRE | MEDI method based on (J. Liu et al., 2012; T. Liu et al., 2011, 2013) | | |  |  |
| Costagli et al., (2016) | ALS | 7T | 0.5×0.5×1 | 5.6 | 6.4 | 7 | GRE | Laplacian-based | V-SHARP | iLSQR | splenium of the corpus callosum |  |
| Lee et al., (2017) | ALS | 3T | 0.6×0.6×2 | 17 | 6.2 | 4 | GRE |  | HARPERELLA |  | subcortical WM |  |
| Acosta-Cabronero et al., (2018) | ALS | 3T | 1×1×2 | 20 | NA | 1 | GRE | Laplacian-based | SHARP | nMEDI | not referenced |  |
| Weidman et al., (2019) | ALS/PLS | 3T | slice thickness: 3 | 53 |  | 11 |  |  |  |  | ipsilateral centrum semiovale anterior to the corticospinal tract |  |
| Welton et al., (2019) | ALS | 3T | 0.47×0.47×3 | 12.74 |  |  | GRE (ESWAN) | (MEDI) toolbox (J. Liu et al., 2012) | | | calcarine sulcus |  |
| Donatelli et al., (2019) | ALS | 3T | 0.94×0.94×1 | 13 | 3.4 | 16 | 3D multi-echo T2*-weighted | Laplacian-based | V-SHARP | iLSQR |  |  |
| Contarino et al., (2020) | ALS | 3T | slice thickness: 1.4 | 24 | 3.3 | 7 | GRE |  | V-SHARP | STAR-QSM |  | STI Suite |
| Fritzsch et al., (2014) | WD | 7T | 0.6×0.6×0.8 | 9.76 | 9.43 | 3 | GRE | Laplacian-based | SHARP | SDI | CSF |  |
| Doganay et al., (2018) | WD | 1.5T | 1.14×1.33×2.5 | echo train: 8.3, 16.8, 24.7, 32.7, 40.7 | | | GRE |  | PDF | MEDI | CSF |  |
| Saracoglu et al., (2018) | WD | 1.5T | 0.14×1.33×2.5 | echo train: 8.3, 16.8, 24.7, 32.7, 40.7 | | | GRE |  | PDF | MEDI | CSF |  |
| Dezortova et al., (2019) | WD | 3T | 0.8×0.8×2 | 5.22 | 5.88 | 6 | GRE | Laplacian based | V-SHARP | nMEDI | occipital white matter |  |
| Domínguez et al., (2016) | HD | 3T | 0.45×0.45×1.5 | 23 | NA | 1 | GRE | Laplacian-based | V-SHARP | LSQR | lateral ventricle CSF |  |
| Van Bergen et al.,(2016) | pre-HD | 7T | 1×1×1 | 4 | 2 | 8 (5 echoes from 10-18 ms TE were used) | GRE | Laplacian-based | V-SHARP | LSQR-based | lateral ventricle CSF |  |
|  | pre-HD |  | 1×1×1 | 2 | 2 | 22 (5 echoes from 10-18 ms TE were used) |  |  |  |  |  |  |
| Chen et al., (2018) | HD | 7T | 1×1×1 | 4 | 2 | 8 last 5 echoes used) | GRE | Laplacian-based | V-SHARP | modified SFCR | CSF in the frontal and body parts of the lateral ventricles |  |
| Harding et al., (2016) | FRDA | 3T | 0.9×0.9×0.9 | echo train: 7.38, 22.14 | | | GRE |  |  |  | frontal white matter | STI suite |
| Ward et al., (2019) | FRDA | 3T | 0.9×0.9×0.9 | echo train: 7.38, 22.14 | | | GRE | Laplacian-based | V-SHARP | iLSQR | whole brain average | STI-Suite v2.2 |
| Xie et al., (2019) | SCA3 | 3T | 0.58×0.94×2 | 5.67 | 6.5 | 8 | GRE (ESWAN) | Laplacian-based | V-SHARP | STAR-QSM | CSF | STI Suite V3.0 |
| Sugiyama et al., (2019) | SCA6 | 3T | 1×1×2 | 6.5 | 6.4 | 7 | GRE | Based on MEDI sequence (J. Liu et al., 2012) | | |  |  |
| Ates et al. (2019) | MD | 3T | 1×1×1.2 | 4.6 | 8 | 3 | 3D fast field echo sequence |  | V-SHARP | HEIDI | whole brain average |  |
| Russo et al. (2018) | FD | 3T | 0.5×0.5×1 | echo train: 7.38, 22.14 | | | GRE |  |  | iLSQR |  |  |
| Dusek et al., (2014) | PKAN | 7T | 0.5×0.5×1 | 15 | NA | 1 | GRE | “Susceptibility calculations using a regularized inverse filter were performed in the selected 3D regions of the Fourier transform of the high-pass-filtered phase image (Haacke et al., 2010)” | | | occipital white matter |  |
| Zeng et al., (2019) | PKAN | 3T | 0.75×0.83×2 | 16 |  |  | SWI |  |  | LSQR |  |  |
| Dusek et al., (2019) | MPAN | 7T | 0.3×0.3×1 | 15.3 |  |  | GRE |  | MSDI | | parietooccipital WM rostral to optic radiation | QSMbox software package |
| CSF: cerebrospinal fluid  EPI: echo planar imaging  ESWAN: enhanced susceptibility-weighted angiography  FFE: fast field-echo  GRE: gradient (recalled) echo  HARPERELLA: harmonic phase removal with the Laplacian operator  HEIDI: homogeneity enabled incremental dipole inversion  iLSQR: iterative LSQR  JEDI: joint background-field removal and segmentation-enhanced dipole inversion  LSQR: sparse linear equation and least-squares  MEDI: morphology enabled dipole inversion  MP-QSM: magnetization-prepared spoiled turbo multiple gradient echo sequence with inversion pulse for QSM  MSDI: multi-scale dipole inversion  MUDICK: multiple dipole-inversion combination with k-space segmentation  nMEDI: non-linear MEDI  PDF: projection onto dipole fields  RE-SHARP: Regularization-enabled SHARP  SDI: superfast dipole inversion  SFCR: structural feature‐based collaborative reconstruction algorithm  SHARP: Sophisticated Harmonic Artifact Reduction for Phase  STAR-QSM: streaking artifact reduction for QSM  SWAN: susceptibility weighted angiography  SWI: susceptibility weighted imaging  TKD: truncated k-space division  TSVD-SHARP: truncated singular value decomposition SHARP  V-SHARP: Variable-radius SHARP | | | | | | | | | | | | |
